# Chalcogen heteroatom engineering of solid additives with optimized excited-state properties enables the development of high-performance organic solar cells

**DOI:** 10.1039/d6sc04665g

**Published:** 2026-09-11

**Authors:** You-Dan Zhang, Xue Chen, Shun Zhang, Huan-Tao Xu, Rong-Rong Liu, Xin-Ru Gao, Haojie Du, Yuhan Song, Cheng-Long Wang, Chunfeng Zhang

**Affiliations:** a National Green Coating Equipment and Technology Research Centre, Lanzhou Jiaotong University Lanzhou 730070 China zyd090211@163.com 18794033064@163.com 18219646379@163.com 1972437478@qq.com clwang@lzjtu.edu.cn; b Lanzhou Longneng Electric Power Science & Technology Ltd Lanzhou 730070 China zhshnet@163.com; c National Laboratory of Solid State Microstructures, Collaborative Innovation Center of Advanced Microstructures, Nanjing University Nanjing 210093 China cfzhang@nju.edu.cn 643266380@qq.com; d School of Environmental and Municipal Engineering, Lanzhou Jiaotong University Lanzhou 730070 China 2060259525@qq.com 2727729930@qq.com

## Abstract

Solid additives (SAs) attract considerable research interest due to their remarkable ability to improve the power conversion efficiency (PCE) of organic solar cells (OSCs). In spite of this, current research on how chalcogen heteroatom engineering of SAs affects the properties of excited states and thereby the performance of OSCs remains relatively scarce. In this work, two simple, halogen-free and low-cost SAs, benzofuran (BFZ) and 2,1,3-benzothiadiazole (BTD), are innovatively selected to conduct a systematic investigation into the influences of chalcogen heteroatom engineering of SAs on the properties of excited states and the performance of OSCs. The femtosecond transient absorption (fs-TA) measurements demonstrate that the BTD-based films exhibit faster hole transfer (HT), longer lifetime and greater quantity of intra-moiety delocalized excited states (*i*-DEs), as well as less non-radiative recombination mediated by triplet states. Among them, the optimal performance is attained in the BTD-treated devices, yielding a PCE of up to 19.11%, significantly superior to that of the control devices (18.12%). This study not only expands the library of halogen-free SAs but also highlights the significance of chalcogen heteroatom engineering of SAs in regulating the properties of excited states, suppressing triplet-mediated energy loss as well as advancing the performance of OSCs.

## Abstract 2

Chalcogen heteroatom engineering of solid additives is a key strategy to regulate excited states, promote charge transport and separation, suppress recombination, thus enhancing photovoltaic performance.

## Graphical abstract

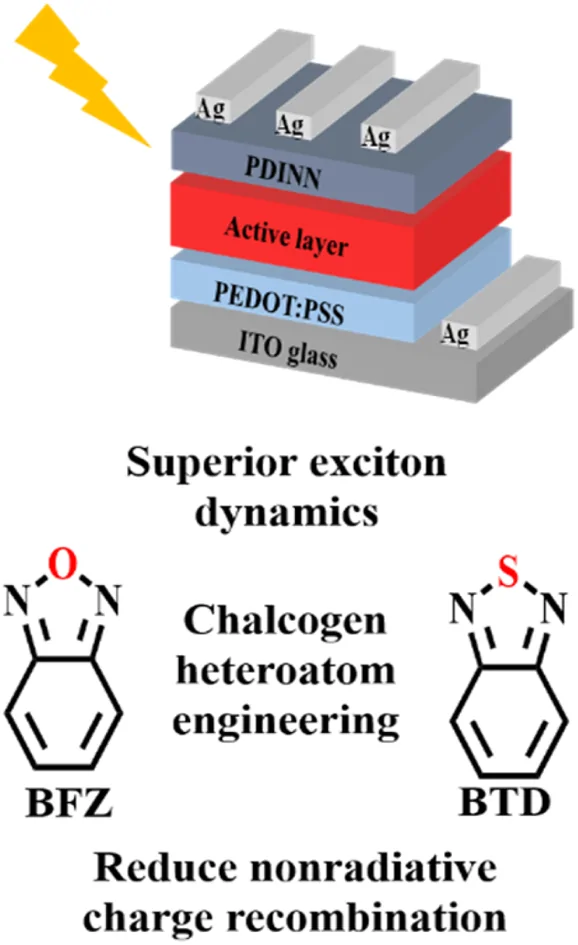

## Introduction

Organic solar cells (OSCs) have attracted growing research interest in the area of sustainable energy due to their merits of flexibility, light weight, low-cost and semi-transparency.^1–5^ In recent decades, through the synergistic optimization of novel materials and device engineering, the power conversion efficiency (PCE) of OSCs has been propelled to approach 21%.^6–12^ Generally, strategies for enhancing the PCE include the synthesis of novel donor (D) and acceptor (A) materials,^13^ optimization of device fabrication processes,^14^ the incorporation of additives and so on.^15^ Among these, additives are widely used due to their simple operation and remarkable effect. At present, solvent additives and solid additives (SAs) represent the two primary categories of established additives. Solvent additives, including 1,8-diiodooctane (DIO) and 1-chloronaphthalene (1-CN), suffer from issues including residual-induced stability degradation and high sensitivity to the amount of addition, which affect the performance of OSCs and the long-term stability.^16^

Given these challenges, SAs have emerged as a research hotspot attributed to their dosage insensitivity, effective avoidance of residual solvent contamination, simple molecular design and so forth.^17^ Meanwhile, the molecular packing is effectively modulated by the intermolecular interactions between SAs and donor or/and acceptor, facilitating exciton dissociation while mitigating charge recombination, thereby improving the performance of OSCs.^18–20^ At the present stage, the most popular and effective SAs usually contain heteroatoms. The halogen-based additives have been extensively studied,^21,22^ whereas the research on chalcogen-based additives remains relatively scarce. Li *et al.* prepared three halogen-free additives *via* chalcogen heteroatom engineering (O → S → Se). Due to the differences in electronegativity and atomic radius of chalcogen atoms, the three additives exhibit distinct dipole moments, which further influence the intermolecular interactions and morphology in the active layer. Consequently, the PM6:L8-BO-based devices modified with S-PA achieved the highest PCE of 18.51%, providing a chalcogen heteroatom engineering strategy for the design of halogen-free additives.^23^ In addition, Liu *et al.* designed three Se-based additives. The introduction of Se-based additives could effectively lengthen *L*_D_, and enhance exciton dissociation and charge collection, while reducing bimolecular and trap-assisted recombination. Consequently, the performances of OSCs including the three additives all outperform the control device, confirming the effectiveness of chalcogen-based additives in enhancing the performance of OSCs.^24^ The great significance and potential of chalcogen engineering of SAs have compelled us to urgently uncover the mechanism of how it regulates the performance of OSCs.

With the development of advanced characterization methods and in-depth insights in this field, researchers have revealed that the properties of excited states have been widely acknowledged to be one of the most intrinsic factors that affect device performance.^21,25–27^ The properties of excited states also influence non-radiative recombination, which has been considered one of the key challenges hindering the further development of OSCs.^28^ Specifically, the non-geminate recombination of free charges, dissociated from localized excitons (LEs) mediated by charge-transfer (CT) states, forms singlet CT (^1^CT) or triplet CT (^3^CT) states at the D/A interface. Typically, the CT state lies energetically higher than the triplet exciton (T_1_) state of either the donor or the acceptor, which results in the relaxation to the T_1_ state and inevitably causes photocurrent loss. Therefore, tailoring the properties of excited states can effectively minimize the relaxation pathway from ^3^CT to T_1_ and suppress triplet-induced energy loss. Even so, there is little research on the properties of excited states and performance of OSCs regarding the impact of chalcogen heteroatom variation in SAs.

In this study, two simple, halogen-free and low-cost SAs, benzofuran (BFZ) and 2,1,3-benzothiadiazole (BTD), were incorporated into PM6:L8-BO binary OSCs by chalcogen heteroatom engineering. The structures of the two SAs are illustrated in Fig. 1a, with varying oxygen (O) and sulfur (S) atoms. The effects of different chalcogen heteroatoms on intermolecular interactions, properties of excited states, recombination process and the final performance of devices were researched systematically and in detail. It was found that the two SAs could form strong intermolecular interactions with L8-BO due to the homologous structure with the center of L8-BO, which favors effectively suppressing the excessive aggregation of L8-BO.^22^ The variation of atomic radius and electronegativity results in different aggregation behaviors and microscopic morphology, thus affecting the properties of excited states and performance of OSCs.^29–31^

**Fig. 1:**
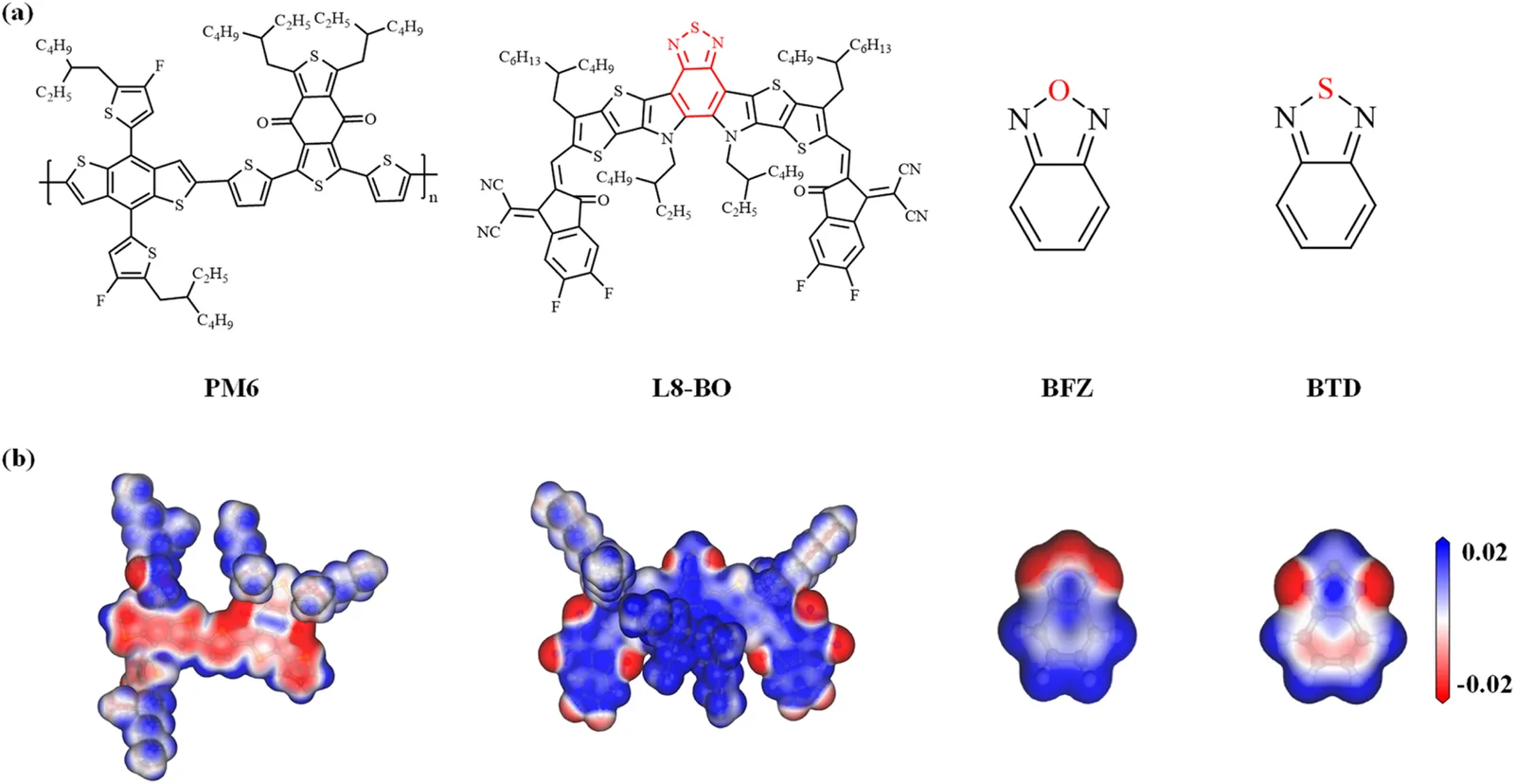
(a) Molecular structures of PM6, L8-BO, BFZ and BTD. (b) ESP distributions for PM6, L8-BO, BFZ and BTD.

Based on these findings, the BTD-treated films exhibited well-defined fibrillar networks and more ordered molecular arrangement by morphological characterization studies, which were in favor of directional transfer of charge and reduction of interface recombination. The results of femtosecond transient absorption (fs-TA) measurements indicated that the faster hole transfer (HT), longer lifetime and greater quantity of intra-moiety delocalized excited states (*i*-DEs), as well as less non-radiative recombination mediated by triplet states could be detected in BTD-based films in comparison with BFZ-based and the control films. Accordingly, BTD-based devices achieved remarkable PCEs of 19.11%, significantly higher than that of BFZ-based (19.04%) and the control devices (18.12%). What's more, BFZ and BTD show a certain universality in other active layers, demonstrating the significant potential in improving the performance of OSCs. This study also highlights the significance of chalcogen heteroatom engineering of SAs in regulating the properties of excited states, suppressing triplet-mediated energy loss as well as advancing the performance of OSCs.

## Results and discussion

Fig. 1a illustrates the structures of PM6, L8-BO, and the SAs. BFZ and BTD are halogen-free as well as low-cost. To examine the miscibility among BFZ, BTD and the active layer, the measurement of contact angles (CAs) was carried out. As presented in Fig. S1 and Table S1 (SI), the surface energies (*γ*) of the neat films were measured as follows: 19.43 mN m^−1^ for PM6, 26.24 mN m^−1^ for L8-BO, 71.23 mN m^−1^ for BFZ and 49.88 mN m^−1^ for BTD. The Huggins parameter (*χ*) serves as an indicator of material miscibility, where a lower value reflects greater compatibility. The *χ* values between PM6 and BFZ and between PM6 and BTD were 16.25 K and 7.05 K, respectively. In comparison, the *χ* values between L8-BO and BFZ and between L8-BO and BTD were 11.00 K and 3.76 K, respectively. The findings indicate that L8-BO and BTD exhibit superior miscibility and stronger intermolecular interaction.

To further investigate the interactions between SAs and the active layer, computations at the B3LYP/6-31G(d) level of theory were performed to obtain the electrostatic potential (ESP) and optimized geometries of PM6 and L8-BO. As illustrated in Fig. 1b, PM6 possesses negative charge distributions in the backbone and positive charge distributions in the alkyl chains, whereas the distribution of L8-BO exhibits predominantly positive charge distribution. There are significant differences between BTD and BFZ in the chalcogen heteroatoms which originate from the intrinsic properties of O and S. Specifically, the atomic radius of S (102 pm) is larger than that of O (73 pm), which makes it more susceptible to being polarized and more favorable to the non-covalent intermolecular interactions, thereby facilitating the ordered molecular arrangement.^29^ Furthermore, the lone pair electrons of S and O can overlap with the delocalized π-orbitals of the adjacent benzene rings to form p–π conjugation. Besides, the 3p orbital of S possesses greater spatial extension compared to the 2p orbital of O, resulting in a stronger p–π conjugated system in BTD.^30^ The enhanced p–π conjugation strengthens the degree of electron delocalization, enhances the light-harvesting capability and facilitates the carrier transport.^32^ Additionally, the electronegativity of S (2.58) is more similar to that of carbon (C) (2.55) compared with that of O (3.44), which leads to a more uniform electron distribution in the S–C bonds, contributing to the formation of a more coherent and delocalized conjugated system, thereby modulating their interactions with the active layer.^31^

Fig. S2a and 2a display the ultraviolet-visible (UV-vis) absorption spectra of PM6 and L8-BO pure films. The normalized blend films were processed *via* two SAs to investigate the influence of SAs on the aggregation behavior of molecules in the active layer. All blend films treated with BFZ and BTD exhibited a distinct red shift, indicating that the SAs caused the active layer to pack more closely, especially BTD-based films. Generally, high stability in OSCs is largely dependent on the effective removal of residual additives from the active layer. To guarantee the effective removal of these two SAs following the annealing process, their volatility was evaluated. Fig. S2b and c show a comparison of the UV-vis absorption spectra of BFZ and BTD pure films under pre-annealing and post-annealing conditions. Clearly, the feature absorption of BFZ and BTD vanished after annealing, which showed that both SAs could be completely removed after annealing. As shown in Fig. S2d, BFZ and BTD were placed on a silicon wafer and kept at 100 °C for a certain time to track their states. It can be clearly observed that BFZ and BTD underwent a phase transition from a solid to a liquid within 5 s and completely volatilized after 10 min.^33^ These results indicate that BFZ and BTD can volatilize completely to avoid residue.

**Fig. 2:**
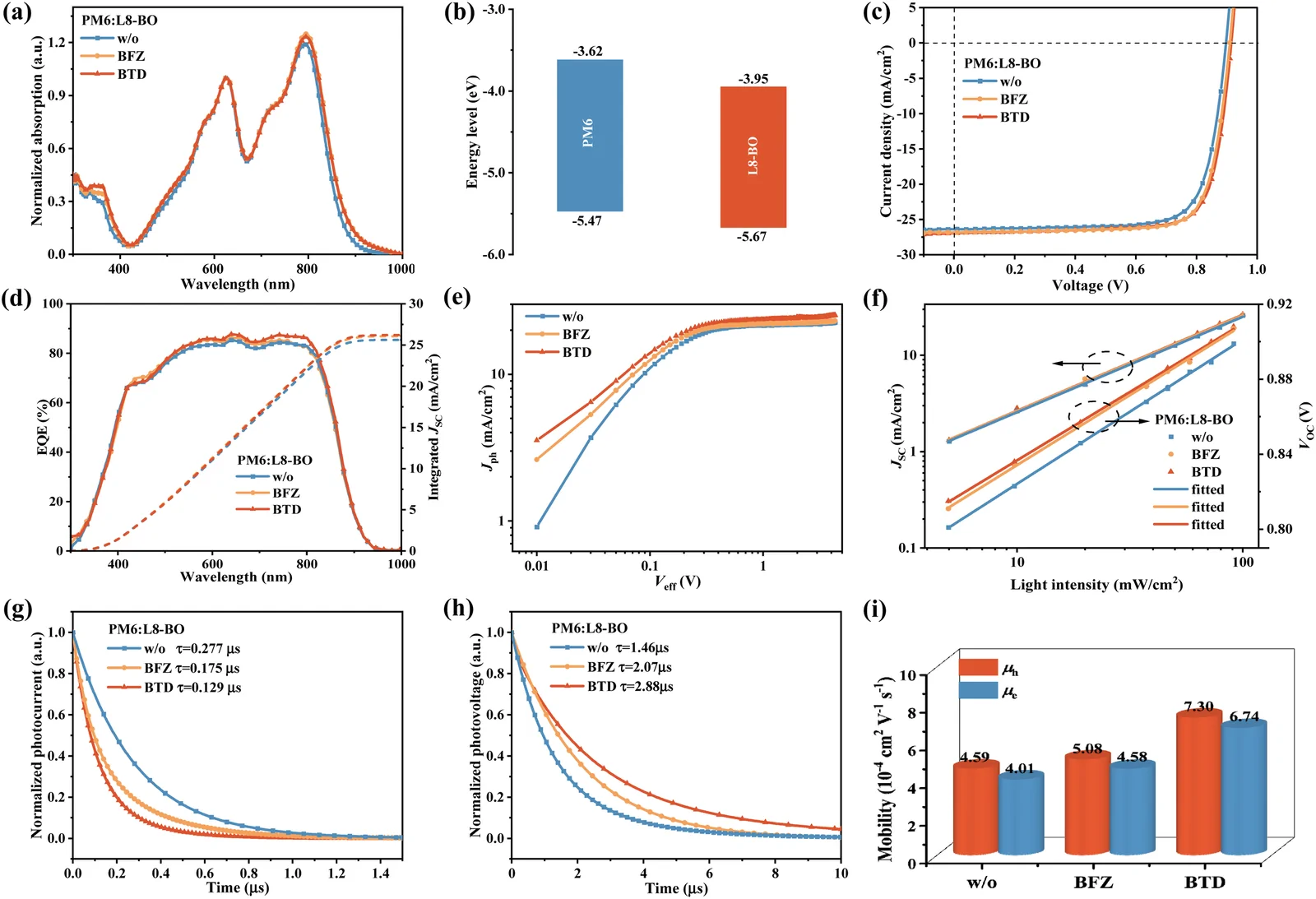
(a) Normalized UV-vis absorption spectra of the blend films. (b) Energy levels of PM6 and L8-BO. (c) *J*–*V* characteristic curves. (d) EQE response curves. (e) *J*_ph_–*V*_eff_ curves. (f) *J*_SC_–*P*_light_ and *V*_OC_–*P*_light_ curves of the control device, and devices with BFZ and with BTD. (g) Transient photocurrent and (h) transient photovoltage measurements. (i) Mobility values.

Fig. 2b and S2e present the cyclic voltammetry (CV) results to determine the energy levels of PM6 and L8-BO. The highest occupied molecular orbital (HOMO) and lowest unoccupied molecular orbital (LUMO) energy levels of PM6 and L8-BO were measured to be −5.47/−3.62 eV and −5.67/−3.95 eV, respectively. Faster charge transfer is promoted by energy cascade between PM6 and L8-BO.

To gain insight into how BFZ and BTD affect the performance of OSCs, devices were fabricated with the architecture of ITO/PEDOT:PSS/active layer/PDINN/Ag, which are described in detail in the SI. To investigate the effect of the additive concentrations on the performance of OSCs, a series of devices was fabricated with the concentrations varying from 0 wt% to 100 wt%. The current density–voltage (*J*–*V*) characteristic curves of the photovoltaic devices with varying additive concentrations are presented in Fig. S3, and the corresponding photovoltaic performance parameters are summarized in Tables S2 and S3. The results indicate that, in the PM6:L8-BO system, the optimal additive content for both BFZ and BTD is 96 wt%. Fig. 2c displays the *J*–*V* plot of the OSCs. Table 1 summarizes the key values of the optimal devices. The control device delivered a PCE of 18.12%, with an open-circuit voltage (*V*_OC_) of 0.900 V, a short-circuit current density (*J*_SC_) of 26.36 mA cm^−2^ and a fill factor (FF) of 76.58%. Upon introducing BFZ and BTD into the films at their optimum concentration of 96 wt%, the resulting devices achieved improved PCEs of 19.04% and 19.11%, respectively. These devices exhibited *V*_OC_ values of 0.910 and 0.915 V, along with *J*_SC_ values of 26.83 and 26.91 mA cm^−2^, and FF values of 77.99% and 77.64%, respectively. The increase in *J*_SC_ can be ascribed to the superior structural ordering and enhanced absorption intensity observed in the devices incorporating BFZ and BTD. BTD was also incorporated into different systems to evaluate the universality (Fig. S4, S5 and Table S4). The external quantum efficiency (EQE) spectra of the three devices are displayed in Fig. 2d. The BFZ- and BTD-based devices exhibit enhanced photon-response in the wavelength range from 450 nm to 800 nm, which is in agreement with absorption spectral trends. The enhanced EQE responsivity confirms the improvement in photocurrent. The calculated integral current (*J*^cal^_SC_) is in good agreement with the *J*_SC_ extracted from the *J*–*V* measurement, verifying the validity of the *J*–*V* measurement.

**Table 1:** Detailed photovoltaic parameters of PM6:L8-BO OSCs

PM6:L8-BO	*V*_OC_ (V)	*J*_SC_ (mA cm^−2^)	*J* ^cal^_SC_ (mA cm^−2^)	FF (%)	PCE (ave. PCE)^a^ (%)
w/o SAs	0.900	26.36	25.62	76.58	18.12 (18.04 ± 0.20)
BFZ	0.910	26.83	26.11	77.99	19.04 (18.78 ± 0.18)
BTD	0.915	26.91	26.31	77.64	19.11 (18.83 ± 0.17)

a The average values with standard deviation were calculated from 10 devices.

The dependence of photocurrent density (*J*_ph_) on the effective voltage (*V*_eff_) was analyzed to probe the impact of SAs on the charge generation efficiency processes, as can be seen in Fig. 2e and Table S5. At *V*_eff_ = 2 V, *J*_ph_ reaches saturation (*J*_sat_) in all devices. This *J*_sat_ value enables calculation of the exciton dissociation probability (*P*_diss_ = *J*_ph_/*J*_sat_) under short-circuit conditions and the charge collection efficiency (*P*_coll_ = *J*_max_/*J*_sat_) at the maximum power point. From the *J*_ph_–*V*_eff_ data, *P*_diss_ values are determined to be 95.68% for the control devices, 97.73% for BFZ-based devices, and 98.37% for BTD-based devices, with corresponding *P*_coll_ values of 84.50%, 86.11%, and 89.55%, respectively, implying that the introduction of BFZ or BTD (especially BTD) into the PM6:L8-BO blend films promotes both exciton dissociation and charge collection.

The extent of charge recombination can be assessed from the relationship between *J*_SC_, *V*_OC_ and light intensity (*P*_light_), as can be seen in Fig. 2f and Table S6. The *α* values derived from the power-law equation *J*_SC_ ∝ *P*^*α*^_light_ were 0.991 for the control device, 0.994 for the BFZ-based device, and 0.997 for the BTD-based device. The BTD-based device exhibits a slope value closest to 1, suggesting that bimolecular recombination is indeed negligible. Additionally, the control device showed a slope of 1.26 *k*_B_*T*/*q* in the *V*_OC_ ∝ *k*_B_*T*/*q* ln *P*_light_ relationship, whereas the BFZ- and BTD-based devices displayed smaller slopes of 1.22 and 1.20 *k*_B_*T*/*q*, respectively, indicating that the incorporation of BFZ and BTD could reduce trap-assisted recombination.

In summary, the incorporation of BFZ and BTD facilitates exciton dissociation and charge collection and suppresses charge recombination in OSCs, resulting in enhanced photovoltaic performance. Transient photocurrent (TPC) and transient photovoltage (TPV) were used to probe the charge generation, transport, recombination and collection behaviors, thereby assessing how BFZ and BTD influence charge extraction and recombination behaviors. As depicted in Fig. 2g and h, under short circuit conditions, faster charge extraction was achieved upon additive incorporation, as evidenced by the reduced extraction times of 0.175 µs (BFZ) and 0.129 µs (BTD), compared to 0.277 µs for the control device. Furthermore, the carrier lifetimes extracted from monoexponential fitting increased from 1.46 µs for the control device to 2.07 µs and 2.88 µs for the BFZ- and BTD-based devices, respectively, suggesting suppressed charge recombination.

Space charge limited current (SCLC) characterization studies enable the evaluation of how BFZ and BTD affect charge mobility and transport balance. The control device shows a hole mobility (*µ*_h_) of 4.59 × 10^−4^ cm^2^ V^−1^ s^−1^ and an electron mobility (*µ*_e_) of 4.01 × 10^−4^ cm^2^ V^−1^ s^−1^, corresponding to a *µ*_h_/*µ*_e_ ratio of 1.14, as summarized in Fig. 2i, S6 and Table S7. Upon introduction of BFZ and BTD, improved *µ*_h_ and *µ*_e_ are obtained. The BFZ-based device delivers a *µ*_h_ of 5.08 × 10^−4^ cm^2^ V^−1^ s^−1^, a *µ*_e_ of 4.58 × 10^−4^ cm^2^ V^−1^ s^−1^ and a *µ*_h_/*µ*_e_ ratio of 1.11, whereas the BTD-based device delivers a *µ*_h_ of 7.30 × 10^−4^ cm^2^ V^−1^ s^−1^, a *µ*_e_ of 6.74 × 10^−4^ cm^2^ V^−1^ s^−1^ and a *µ*_h_/*µ*_e_ ratio of 1.09. BTD-based devices exhibit higher and more balanced mobility than BFZ-based devices and the control. This might be attributed to the fact that BTD-based devices can effectively suppress carrier recombination, prolong carrier lifetime, promote efficient exciton dissociation and reduce the formation of charge trap states. These results account for the improved FF and *J*_SC_ observed in BTD-based devices.

The ordered crystallinity of the blend films with the incorporation of BFZ and BTD is investigated through a series of morphological analyses, including atomic force microscopy (AFM), transmission electron microscopy (TEM) and grazing-incidence wide-angle X-ray scattering (GIWAXS), as presented in Fig. 3 and S7. As illustrated in Fig. 3a–c, the root-mean-square (RMS) roughness values of 1.178 nm, 0.960 nm and 0.876 nm are obtained for the control, BFZ-based and BTD-based films, respectively. The results demonstrate that the addition of two SAs to the blend films induces formation of a fibrous-network structure, accompanied by a reduction in RMS roughness. The results indicate that the BTD-based films exhibit a more well-defined 3D interpenetrating network and phase separation, which can be ascribed to the enhanced crystallization and enhanced intermolecular interactions in the BTD-based films. This conclusion is also confirmed from the TEM (Fig. S7) images.

**Fig. 3:**
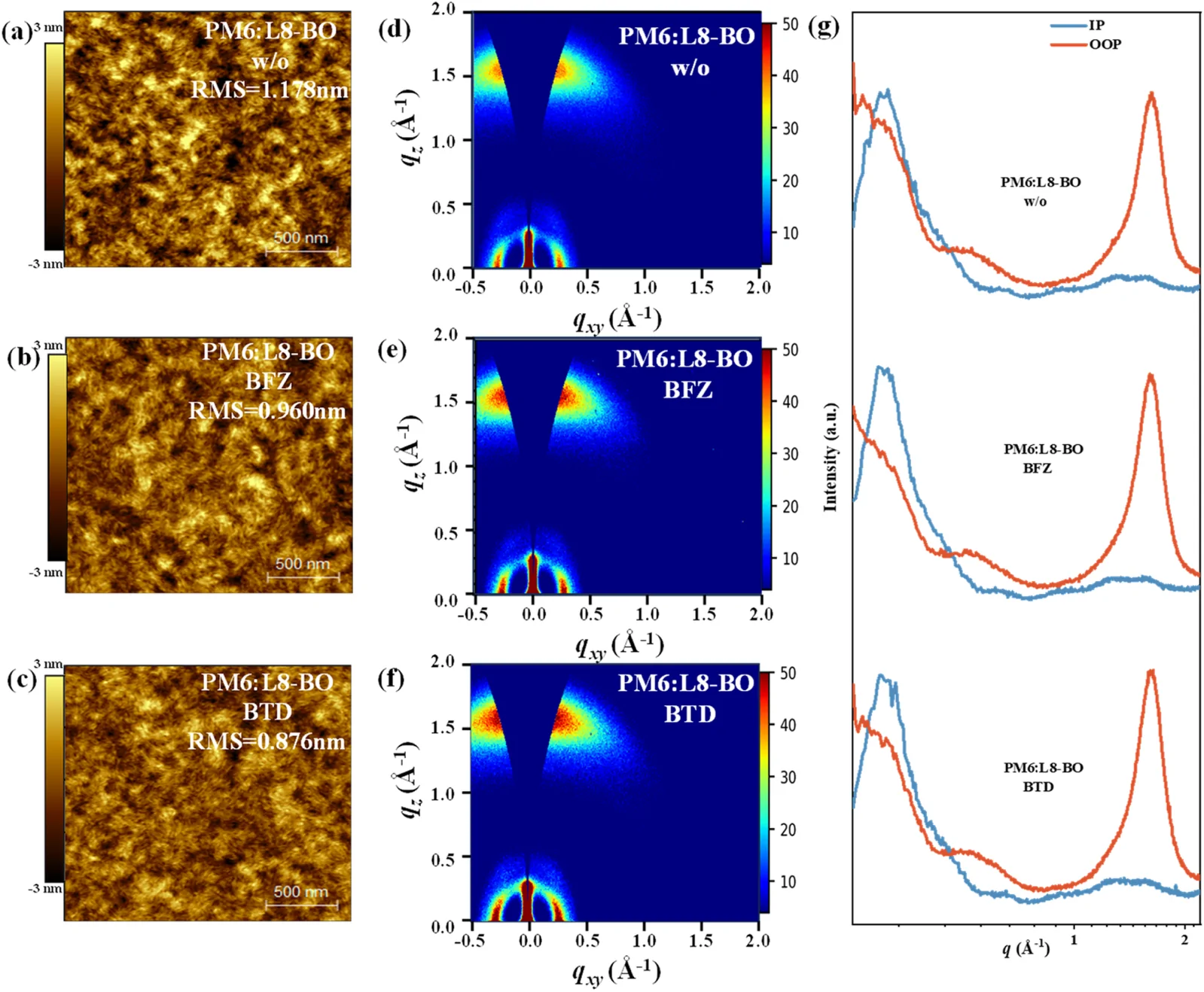
AFM images of PM6:L8-BO blends: (a) w/o SAs, (b) with BFZ and (c) with BTD. Corresponding 2D GIWAXS patterns of PM6:L8-BO blends: (d) w/o SAs, (e) with BFZ and (f) with BTD. (g) OOP and IP line-cut profiles of the GIWAXS patterns of PM6:L8-BO blends.

Further studies were carried out to investigate the influence of SAs on molecular packing and ordering by GIWAXS measurements. Fig. 3d–g present the 2D-GIWAXS diffraction patterns along with their corresponding 1D line cuts. All three blend films exhibit typical face-on oriented stacking, as demonstrated by the (010) diffraction peak along the out-of-plane (OOP) direction and the (100) diffraction peak along the in-plane (IP) direction, in favor of charge transport along vertical direction. For the control films, a π–π stacking peak was observed at *q*_*z*_ = 1.61 Å^−1^, corresponding to a π–π stacking distance of 3.91 Å and a crystal coherence length (CCL) of 22.45 Å. Meanwhile, it showed a lamellar stacking peak at *q*_*xy*_ = 0.31 Å^−1^, corresponding to a lamellar stacking distance of 20.47 Å and a CCL of 57.68 Å. After incorporating BFZ and BTD into the blend, the π–π stacking peaks in the *q*_*z*_ direction were observed at 1.61 Å^−1^ and 1.62 Å^−1^, respectively. This results in reduced π–π stacking distances of 3.90 Å and 3.88 Å, along with increased CCL values of 23.72 Å and 24.45 Å, respectively, as summarized in Tables S8 and S9. In the case of the IP direction, the peaks in the *q*_*xy*_ direction were all observed at 0.31 Å^−1^ after incorporating BFZ and BTD into the blend. These shortened the lamellar stacking distances to 20.44 Å and 20.40 Å, and raised the CCL values to 63.80 Å and 64.24 Å, respectively. Consequently, the BTD-based films exhibit the maximum CCL values for both π–π stacking and lamellar stacking, a characteristic that promotes efficient charge transport. These findings demonstrate that the addition of SAs facilitates precise regulation of molecular packing and crystallization behavior.^34^

To quantitatively analyse the effects of SAs containing different chalcogen heteroatoms on the dynamics of exciton/charge generation, separation, and recombination, we performed fs-TA spectroscopy characterization studies. To reduce disturbance of exciton–exciton annihilation (EEA), we used a low energy of approximately 2 µJ cm^−2^. The corresponding 2D pseudo-color fs-TA spectra are demonstrated in Fig. 4a–f and Fig. S8 displays the spectra at various delay times.

**Fig. 4:**
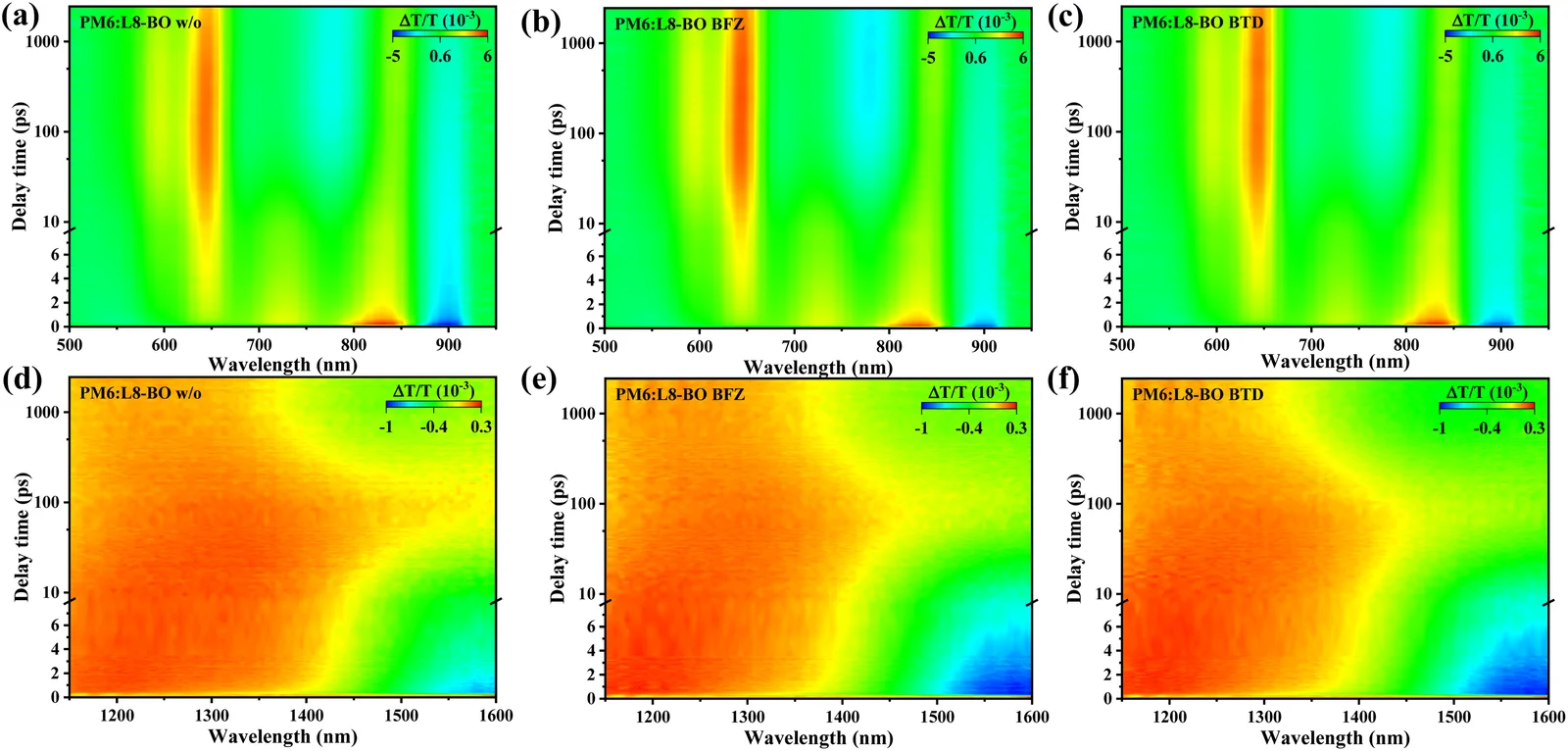
2D color plots of TA spectra of blend films (a and d) w/o SAs, (b and e) with BFZ and (c and f) with BTD.

To investigate the regulatory effects of the two SAs on exciton behavior, the exciton diffusion lengths (*L*_D_) were measured using fs-TA under different pump fluences, and the corresponding results are presented in Fig. S9 and Table S10 (see the SI for the detailed method). The *L*_D_ values were calculated according to the equation *L*_D_ = (*Dτ*)^1/2^, where *D* is the exciton diffusion coefficient and *τ* is the exciton lifetime.^35^ The *L*_D_ values of the L8-BO films processed with BFZ/BTD were determined to be 24.1/26.7 nm, higher than that of the control films (18.7 nm), which means that excitons generated away from the interface have a higher probability of reaching the interface before recombination.

In order to investigate the HT process with different blend films, a pump wavelength of 800 nm was selected to selectively excite the acceptor. Upon excitation, the positive signals centered at about 830 nm could be assigned to the ground state bleaching (GSB) of L8-BO. Along with the fast decay of the GSB signal of L8-BO, the peaks near 630 nm are assigned to the GSB of PM6. This phenomenon arises from the rapid HT process from L8-BO to PM6, coupled with the efficient exciton dissociation in the active layer. Normalized HT dynamic profiles of the three blend films are presented in Fig. S10a. To analyze the HT dynamics *via* fitting with a biexponential function, the lifetimes are *τ*_1_ = 0.38 ps (*A*_1_ = 0.24) and *τ*_2_ = 21.44 ps (*A*_2_ = 0.51) in the control device, *τ*_1_ = 0.19 ps (*A*_1_ = 0.39) and *τ*_2_ = 19.81 ps (*A*_2_ = 0.57) in the BFZ-based device and *τ*_1_ = 0.13 ps (*A*_1_ = 0.67) and *τ*_2_ = 17.35 ps (*A*_2_ = 0.57) in the BTD-based device (Table S11). Usually, *τ*_1_ and *A*_1_ represent the rapid charge separation at the interface and relative amplitude, respectively, while *τ*_2_ and *A*_2_ refer to the exciton diffusion within the domain and relative amplitude, respectively.^36^ By comparing the fitting relative amplitudes, it can be concluded that a larger proportion of excitons underwent ultrafast dissociation directly at the D/A interface upon photoexcitation after the introduction of BTD, thereby promoting more efficient and faster HT at the interface. In addition, compared with O in BFZ, the larger atomic radius of S in BTD, its closer electronegativity to that of C and the more extended 3p orbitals are in favor of the more delocalized conjugated system and stronger orbital hybridization. These contribute to more ordered molecular packing in the BTD-based films, thereby enhancing electronic coupling (*V*) at the D/A interface.^37,38^ According to Marcus theory, the interfacial HT rate (*k*_HT_) is strictly proportional to the square of *V* (*k*_HT_ ∝ |*V*|^2^);^39,40^ combined with the lengthening of *L*_D_, this results in the acceleration of the overall HT dynamics.

As depicted in Fig. 4a–c, a distinct negative signal centered at 902 nm was observed, corresponding to the excited-state absorption (ESA) of LEs. As exhibited in Fig. 4d–f, with the decay of the ESA signal from LEs and the attenuation of coulombic interactions, a more distinct and longer-lived species emerged in the region of 1200–1600 nm, peaking at approximately 1550 nm. Consistent with previous reports, these species were identified as *i*-DEs.^41^

Typically, the *i*-DE is capable of directly yielding free charge carriers without the intervention of interfacial CT states. In the process of charge transport and extraction, free charges may encounter each other again at the D/A interface to form CT states, which statistically possess 75% triplet character (^3^CT) and can relax to T_1_ excitons, causing irreversible non-radiative losses. However, in the BTD-based film, the enlarged and ordered domain sizes reduce the number of D/A interfaces where non-geminate recombination occurs, thereby suppressing the probability of non-radiative recombination mediated by T_1_ formation induced by CT states.^42^ Fig. S10b shows the dynamic profiles of *i*-DEs in all blend films. The fitted lifetimes are 13.30 ps in the control device, 16.24 ps in the BFZ-based device and 20.14 ps in the BTD-based device. Under normal conditions, the relatively longer lifetime of the *i*-DE contributes to reducing non-geminate charge recombination during the process of charge transport and extraction, which may reduce the probability of separated free charges encountering again at D/A interfaces and undergoing recombination, thereby alleviating voltage losses.

Along with the decay of the *i*-DE, another new ESA band with a peak at about 770 nm arises, which can be regarded as the prolonged charge separation (CS) state containing free electrons and holes through the HT process. The ESA of the *i*-DE had an obvious blue shift in all blend films after 100 ps in the NIR region, indicating that another new species had formed with the decay of the CS state and that the recombination of free charges led to the formation of a new species.^43^ According to previous related research, the new species were attributed to the ESA of T_1_. At the interfaces, the separated free charges may encounter each other again at the D/A interface to form CT states, including the states with ^1^CT or ^3^CT. Normally, the energy of ^3^CT is higher than that of the T_1_ of the donor or acceptor and will naturally decay to T_1_. Thus, T_1_ has no choice but to further decay to the ground state, irreversibly resulting in the non-geminate charge recombination.^44^ The content of T_1_ gradually decreases in the order of the control films, BFZ-based films, and BTD-based films, as shown in Fig. S10c.

Combined with the results of GIWAXS measurements, the introduction of the two SAs results in the stronger intensity of diffraction peaks, the reduced π–π stacking distances and the increased CCLs, suggesting more ordered molecular packing and enlarged domain sizes. The enlarged and ordered domain sizes reduce the D/A interfaces within mixed amorphous domains and more discrete interfaces to D or A aggregates, thereby suppressing CT states from the non-geminate recombination of carriers.^45^ Meanwhile, the charge dissociation efficiency is maintained. Combined with the results of EQE, SCLC and GIWAXS, the multiple factors result in improved *J*_SC_.

To investigate the intrinsic contributions of the two additives to the improvement in *V*_OC_, the energy loss (*E*_loss_) of the PM6:L8-BO system was finely deconvoluted using the Fourier-transform photocurrent spectra (s-EQE) and electroluminescence (EL) spectroscopy (Fig. 5). The total *E*_loss_ (*E*_loss_ = *E*_g_ − *qV*_OC_) of organic photovoltaic devices can be divided into three components: the radiative loss above the bandgap (Δ*E*_1_), the radiative loss below the bandgap (Δ*E*_2_), and the non-radiative recombination loss (Δ*E*_3_). Among them, Δ*E*_1_ is determined solely by the bandgap of the material and is inevitable for any photovoltaic system. The Δ*E*_1_ values are 0.270 eV for the control devices and 0.268 eV for the BFZ- and BTD-processed devices, indicating that the contribution from the bandgap itself shows no significant difference among the various systems.

**Fig. 5:**
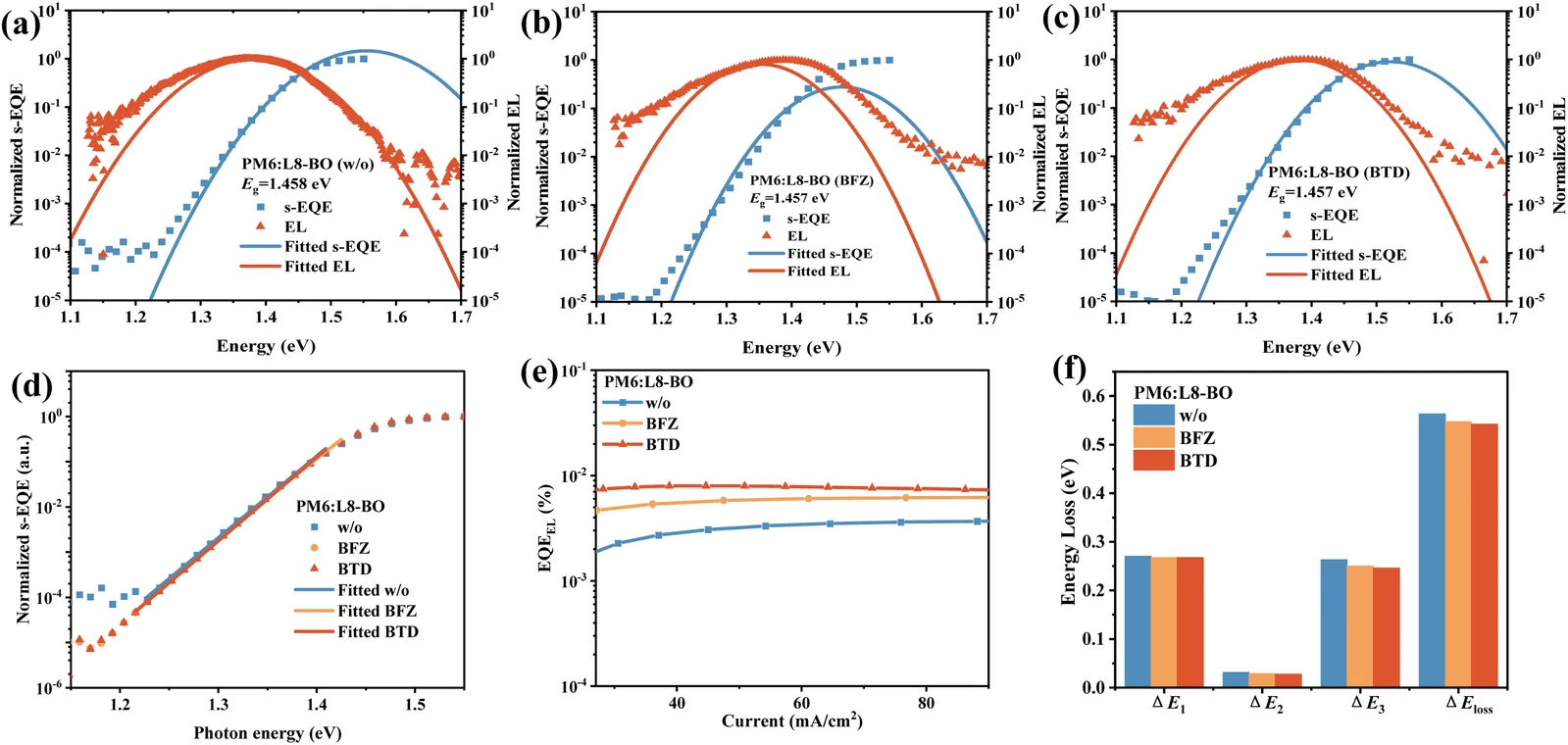
EQE and EL spectra of devices (a) without SAs, (b) with BFZ and (c) with BTD. (d) s-EQE spectra of the untreated and BFZ- and BTD-treated devices. (e) EQE_EL_ curves of the OSCs. (f) Energy loss diagrams of the OSCs.

Regarding Δ*E*_2_, which is generally correlated with the energetic disorder of band tail states, it can be semi-quantitatively evaluated from the Urbach energy (*E*_U_) (Fig. 5d). Upon the introduction of BFZ/BTD, the *E*_U_ value is progressively reduced from 24.05 meV for the control to 23.40/23.19 meV, indicating that the incorporation of SAs could diminish the energetic disorder within the active layer. The non-radiative recombination loss (Δ*E*_3_) is a key factor governing the upper limit of the *V*_OC_ loss, which is typically derived from the electroluminescence external quantum efficiency (EQE_EL_) *via* the relation Δ*E*_3_ = − *k*_B_*T* ln EQE_EL_ (Fig. 5e). For the control devices, the Δ*E*_3_ value is 0.263 eV; upon the addition of BFZ and BTD, the Δ*E*_3_ values decrease to 0.250 and 0.246 eV, respectively, which is consistent with the results of the T_1_ kinetic traces. The detailed data are summarized in Fig. 5f and Table S12. Therefore, the introduction of BTD exhibits a markedly reduced non-radiative voltage loss, resulting in increased *V*_OC_.

The long-term stability of OSCs is a critical parameter for practical applications. The tests for storage stability and thermal stability at 60 °C were conducted in a nitrogen glovebox without encapsulation, with the relative spectra presented in Fig. S11. After 480 hours of continuous aging, the thermal stability was improved from 65.18% for the control devices to 72.26% and 75.20% for the BFZ- and BTD-based devices, respectively, in terms of retaining the initial PCE. Similarly, the storage stability was enhanced from 79.43% to 83.58% and 84.91%, respectively. These results demonstrate that the incorporation of the two SAs can effectively suppress the performance degradation of the devices.

## Conclusions

This study selected two halogen-free SAs, BFZ and BTD, to systematically investigate their effects on the properties of excited states and performance of OSCs *via* chalcogen heteroatom engineering. Compared with O, S has a larger atomic radius, stronger orbital overlap and closer electronegativity to that of C, enabling BTD to form a more delocalized conjugated system. The device based on BTD achieved optimized phase separation, facilitated hole transfer and suppressed triplet-mediated energy loss. Consequently, the BTD-based devices achieve an efficiency of 19.11%, outperforming the BFZ-based (19.04%) and control (18.12%) devices. The universality of SAs was exemplified in several systems. This study verifies the pivotal function of chalcogen heteroatom engineering in achieving high-performance OSCs and highlights its function in regulating the properties of excited states, suppressing triplet-mediated energy loss.

## Author contributions

You-Dan Zhang: writing – review & editing, funding acquisition, supervision. Xue Chen: writing – original draft. Shun Zhang: software. Huan-Tao Xu: methodology. Rong-Rong Liu: formal analysis. Xin-Ru Gao: formal analysis. Haojie Du: investigation. Yuhan Song: investigation. Cheng-Long Wang: supervision. Chunfeng Zhang: project administration, supervision.

## Conflicts of interest

There are no conflicts to declare.

## Supplementary Material

SC-017-D6SC04665G-s001

## Data Availability

The data supporting this article have been included as part of the supplementary information (SI). Supplementary information: detailed information of materials, device fabrication precesses as well as various characterization methods and so on. See DOI: https://doi.org/10.1039/d6sc04665g.

## References

[cit1] Gao C., Wang Y., Tian H., Sun K., Zhao C., Che J., Qiu S., Chen W., Huang C., Wang Z., Hu H., Han P., Li S., Luo Z., Zhang G. (2026). Prolonged-nucleation strategy *via* an asymmetric brominated acceptor enables >20% efficiency in five different organic solar cells. Adv. Mater..

[cit2] Liu L., Zhu H., Wang J., Wang D., Cai D., Wang J., Tu Q., Ma Y., Zheng Q. (2026). Conformational locking through intramolecular F⋯H interactions in dimerized M-series acceptors boosts efficiency and stability of organic solar cells. Energy Environ. Sci..

[cit3] Wang X., Wu J., Zhao S., Chen M., Shi T., Xie X., Bai Q., Xie J., Zhang L., Ma D., Chen J. (2025). Elevating dielectric constant *via* additive engineering: achieving 19.23% certified efficiency in thick-film binary organic solar cells. Joule.

[cit4] Yang Y., Wei L., Zhan L., Liu Y., Lu H., Wu X., Wupur A., Chen T., Yu J., Sun X., Hu H., Sun R., Min J., Luo Y., Wu J., Fu W., Yin S., Chen H. (2025). Ternary strategy and molecular electrostatics collaboratively optimize low-molecular-weight polymer donor organic solar cells: over 20% efficiency and high scalability. Adv. Mater..

[cit5] Zhang Y., Liu R., Zhang B., Chen X., Zhang Y., Wang C., Zhang H. (2025). Simultaneous morphology control and excited-state engineering in organic photovoltaics using volatile solid additives. J. Mater. Chem. A.

[cit6] Fu J., Li H., Liu H., Huang P., Chen H., Fong P., Peña T., Li M., Lu X., Cheng P., Xiao Z., Lu S., Li G. (2025). Two-step crystallization modulated through acenaphthene enabling 21% binary organic solar cells and 83.2% fill factor. Nat. Energy.

[cit7] Li C., Cai Y., Hu P., Liu T., Zhu L., Zeng R., Han F., Zhang M., Zhang M., Lv J., Ma Y., Han D., Zhang M., Lin Q., Xu J., Yu N., Qiao J., Wang J., Zhang X., Xia J., Tang Z., Ye L., Li X., Xu Z., Hao X., Peng Q., Liu F., Guo L., Huang H. (2025). Organic solar cells with 21% efficiency enabled by a hybrid interfacial layer with dual-component synergy. Nat. Mater..

[cit8] Mou H., Yin Y., Chen H., Xu J., Ding J., Ju C., Zhu J., Wang Y., Chen W., Xu G., Zhang T., Li J., Li Y., Li Y. (2025). Transient dipole strategy boosts highly oriented self-assembled monolayers for organic solar cells approaching 21% efficiency. J. Am. Chem. Soc..

[cit9] Shi K., Zhao Q., Liu H., Qin S., Yang S., Mou X., Liang J., Chen J., Hu X., Yao J., Zhu C., Ding W., Zhong L., Guo J., Zhang J., Zhou E., Zhang Z., Yang C., Qiu B., Li Y. (2026). Extended and balanced crystallization kinetics *via* electrostatic potential distribution manipulation of solid additives for organic solar cells reaching 20.89% efficiency. Adv. Energy Mater..

[cit10] Wang Y., Wen J., Zhong Y., Fu L., You Z., Li H., Han G., Han W., Liu J., Zhang H., Feng Y., Li H., Liu W., Zhang J., Han K., Liu Y. (2026). Homology-guided zwitterionic interlayers for 21% efficiency non-fullerene organic solar cells. Adv. Mater..

[cit11] Xu S., Zhang Y., Sun Y., Cheng P., Yao Z., Li N., Ye L., Zuo L., Gao K. (2025). An unprecedented efficiency with approaching 21% enabled by additive-assisted layer-by-layer processing in organic solar cells. Nano-Micro Lett..

[cit12] Yang X., Xie Y., Tian J., Chen J., Zhang Z., Tang D., Shi X., Hao X., Zhang J., Ding L., Sun Y., Lv M. (2026). Achieving a record fill factor of approaching 84% and 21% efficiency in binary organic solar cells *via* solid additive engineering. Adv. Mater..

[cit13] Zhu Y., He D., Kong J., Wang Z., Li B., Chen N., Chai Y., Li X., Zhang Y., Zhang J., He Y., Li Y., Wang C., Zhao F. (2026). Regulating H-/J-aggregation and suppressing exciton–vibration coupling of fused ring electron acceptors enable 20.51% efficiency of organic solar cells. Adv. Mater..

[cit14] Zhou W., Dai X., Fan B., Li H., Xu X., Wu Y., Peng Q. (2025). Solvent vapor diffusion-driven multiscale pre-aggregation of non-fullerene acceptors enables high-performance organic solar cells. Nat. Commun..

[cit15] Shi K., Liu H., Qin S., Zhao Q., Mou X., Liang J., Yao J., Zhu C., Zhong L., Guo J., Zhang J., Wu Y., Zhang Z., Qiu B., Li Y. (2025). Selectively modulating the donor and acceptor aggregation behaviors through solid additive isomerization engineering for organic solar cells exceeding 20% efficiency. Angew. Chem., Int. Ed..

[cit16] Cheng Y., Li H., Zhang X., Chen Y., Ran G., Yue Y., Wei N., Zhu X., Yan X., Liu Y., Lu H., Liu Y., Wei Y., Zhang W., Bo Z. (2025). Enabling high-efficiency and stable binary organic solar cells by solid additive-assisted morphology modulation. Adv. Funct. Mater..

[cit17] Kong L., Zhang Z., Zhao N., Cai Z., Zhang J., Luo M., Wang X., Chen M., Zhang W., Zhang L., Wei Z., Chen J. (2023). *In situ* removable additive assisted organic solar cells achieving efficiency over 19% and fill factor exceeding 81%. Adv. Energy Mater..

[cit18] Huo Y., Cheng B., Kan L., Guo X., Xia X., Han C., Ning S., Hou W., Liu F., Zhang M. (2026). Sequential crystallization engineering *via* a
dual-functional thiophene derivative boosts organic solar cell efficiency to 20.5%. Adv. Funct. Mater..

[cit19] Wang Y., Sun W., Zhang Y., Zhang B., Ding Y., Zhang Z., Meng L., Huang K., Ma W., Zhang H. (2025). Integrated omnidirectional design of non-volatile solid additive enables binary organic solar cells with efficiency exceeding 19.5%. Angew. Chem., Int. Ed..

[cit20] Zhao B., Zhu L., Xiong S., Yu J., Wang X., Zhao J., Tan L., Zhang J., Zhong J., Kan L., Wan X., Jiang K., Li H., Ma Z., Liu Y., Zhu H., Kan Z., Liu F., Sun Z., Chu J., Bao Q. (2025). Boosting binary organic solar cells over 20% efficiency *via* synchronous modulation of charge transport and phase morphology. Adv. Energy Mater..

[cit21] Liao C., Jin W., Zhou W., Deng M., Xu X., Dai L., Peng Q. (2025). Halogen-engineered thiophene additives enable high-performance layer-by-layer organic solar cells with 20.12% efficiency. Carbon Energy.

[cit22] Maqsood M., Chen N., Xiang K., Bai H., Su W., Xin J., Li T., Liu H., Wang K., Shen Y., Zhang D., Liu Y., Ji Y., Bi Z., Li X., Yan L., Chen J., Lu G., Liu Y., Yu D., Gao Z., Zhu W., Ma W., Fan Q. (2026). Halogenated benzothiadiazole solid additives enable 20.12% efficiency layer-by-layer organic solar cells. Adv. Funct. Mater..

[cit23] Li T., Zhu Y., Peng X., Li H., Ye L., Wang Q., Zhang Z., Shen P., Weng C. (2025). Chalcogen heteroatom engineering of non-halogenated volatile solid additives based on phthalic anhydride enables high-performance polymer solar cells. Chem. Eng. J..

[cit24] Liu Z., Fu Y., Zheng W., Wu J., Zhao M., Liu J., Yan H., Xie Z. (2026). Additive-induced fibrillation of acceptor enhances the efficiency of sequentially deposited organic solar cells approaching 20%. Adv. Funct. Mater..

[cit25] Chen T., Zhong Y., Dong X., Wang J., Feng W., Zhang J., Han K., Wupu A., Fu W., Kan B., Chen Y. (2025). A p-type liquid-crystal semiconductor with synergistic morphological and charge-dynamic modulation enables 20.3%-efficiency binary organic solar cells. Adv. Mater..

[cit26] Duan X., Zhang J., Kong J., Song B., Qiao J., Deng M., Li W., Woo H., Hao X., Lu G., Song J., Sun Y. (2026). Over 20.5% efficiency of halogen-free solvent-processed organic solar cells achieved by anti-solvent strategy. Adv. Mater..

[cit27] Zhang Y., Wang X., Fei X., Zhang L., Guo P., Li M., Xia Y., Wang C., Zhang H. (2021). Photodynamic investigation on the synergistic effects of aromatic side chains with alkylthio substituents in nonfullerene organic solar cells. ACS Appl. Energy Mater..

[cit28] Zhang Y., Wang X., Fei X., Li M., Wang C., Zhang H. (2022). Enhanced photodynamic of carriers
and suppressed charge recombination enable approaching 18% efficiency in nonfullerene organic solar cells. ACS Appl. Mater. Interfaces.

[cit29] Cao X., Xu Z., Wang R., Guo J., Zhao W., Zhang Y., Yao Z., Guo Y., Long G., Wan X., Chen Y. (2025). O, S, and N bridged atoms screening on 2D conjugated central units of high-performance acceptors. Adv. Mater..

[cit30] Yang H., Cui C., Li Y. (2021). Effects of heteroatom substitution on the photovoltaic performance of donor materials in organic solar cells. Acc. Mater. Res..

[cit31] Zhou Y., Qi G., Liu H., Bai H., Li T., Maqsood M., Liu C., Song B., Chen N., Lu G., Gao C., Liu Y., Su W., Du H., Ma R., Ma W., Fan Q. (2025). Fluorine/bromine/selenium multi-heteroatoms substituted dual-asymmetric electron acceptors for *o*-xylene processed organic solar cells with 19.12% efficiency. Sci. China Mater..

[cit32] Chen T., Zhong Y., Duan T., Tang X., Zhao W., Wang J., Lu G., Long G., Zhang J., Han K., Wan X., Kan B., Chen Y. (2025). Asymmetrified benzothiadiazole-based solid additives enable all-polymer solar cells with efficiency over 19%. Angew. Chem., Int. Ed..

[cit33] Xiang H., Sun F., Zheng X., Gao B., Zhu P., Cong T., Li Y., Wang X., Yang R. (2024). Tackling energy loss in organic solar cells *via* volatile solid additive strategy. Adv. Sci..

[cit34] Cheng J., Wang L., Wang T., Chen C., Sun Y., Zhou J., Gan Z., Xia W., Gao D., Liu D., Li W., Wang T. (2025). Vacuum-assisted vertical component distribution in pseudo-bulk heterojunctions: a pathway to high-performance and stable organic solar cells. Natl. Sci. Rev..

[cit35] Luo D., Jiang Z., Tan W., Zhang L., Li L., Shan C., McNeill C., Sonar P., Xu B., Kyaw A. (2023). Non-fused ring acceptors achieving over 15.6% efficiency organic solar cell by long exciton diffusion length of alloy-like phase and vertical phase separation induced by hole transport layer. Adv. Energy Mater..

[cit36] Zhou Y., Su W., Liang Z., Wu Q., Bai H., Liu H., Song B., Li X., Xie B., Liu C., Zhang Y., Wang Y., Cao J., Liao X., Lu G., Liu Y., Ma R., Du H., Ma W., Fan Q. (2025). Halogenated diphenyl ether solvent additives enable ∼20% efficiency organic solar cells and high-performance opaque/semitransparent modules. Natl. Sci. Rev..

[cit37] Li Y., Jia Z., Huang P., Gao C., Wang Y., Xue S., Lu S., Yang Y. (2025). Simultaneously improving the efficiencies of organic photovoltaic devices and modules by finely manipulating the aggregation behaviors of Y-series molecules. Energy Environ. Sci..

[cit38] Zhang J., Ma R., Li R., Tang J., Luo D., Chen L., Chen Z., Wang Y., Chen H., Feng J., Zhang G., Yu L., Ye L., Li Y., Li G., Yang C., Luo Z. (2025). Fused-ring isomerism modulates molecular packing and device performance in nonhalogenated organic solar cells. Nat. Commun..

[cit39] Zhong Y., Causa M., Moore G., Krauspe P., Xiao B., Günther F., Kublitski J., Shivhare R., Benduhn J., BarOr E., Mukherjee S., Yallum K., Réhault J., Mannsfeld S., Neher D., Richter L., DeLongchamp D., Ortmann F., Vandewal K., Zhou E., Banerji N. (2020). Sub-picosecond charge-transfer at near-zero driving force in polymer:non-fullerene acceptor blends and bilayers. Nat. Commun..

[cit40] Zhou G., Zhang M., Chen Z., Zhang J., Zhan L., Li S., Zhu L., Wang Z., Zhu X., Chen H., Wang L., Liu F., Zhu H. (2021). Marcus Hole Transfer Governs Charge Generation and Device Operation in Nonfullerene Organic Solar Cells. ACS Energy Lett..

[cit41] Zhou M., Xu X., Wang D., Song J., Qiao J., Zhang R., Wu Y., Wang T., Cui J., Hall C., Yin H., Du X., Qin W., Gao K., Chen F., Wei Z., Ashokkumar M., Smith T., Zhang K., Sun Y., Hao X. (2025). Sonication-induced J-aggregation in nonhalogenated solvents boosts exciton delocalization for high-efficiency organic solar cells. Nat. Commun..

[cit42] Lv J., Fu J., Su W., Ran G., Zheng D., Ding X., Sun X., Wang F., Zhao J., Gao C., Zheng J., Zhang G., Kan Z., Yang C., Wang Z., Ren Z., Li J., Wang H., Zhang W., Li G., Hu H. (2026). Interfacial energy re-excitation enables 20.5% efficiency organic solar cells *via* exciton delocalization. Matter.

[cit43] Zhang Y., Ouyang Y., Liu R., Chen X., Wang C., Wang X., Zhang C. (2025). Manipulating excited-state dynamics to unlock low energy loss for high-performance organic solar cells. ACS Appl. Energy Mater..

[cit44] Guo L., Wu L., Zhang H., Pan Y., Li X., Dong X., Kong J., Jee M., Mai M., Liu S., Woo H., Jia T., Zhao Z., Gao F., Wang Z., Sun Y. (2026). Dimeric acceptor with small singlet–triplet energy gap enables suppressed triplet loss and 20.85% efficiency of organic solar cells. Angew. Chem., Int. Ed..

[cit45] Jiang K., Zhang J., Zhong C., Lin F., Qi F., Li Q., Peng Z., Kaminsky W., Jang S.-H., Yu J., Deng X., Hu H., Shen D., Gao F., Ade H., Xiao M., Zhang C., Jen A. (2022). Suppressed recombination loss in organic photovoltaics adopting a planar-mixed heterojunction architecture. Nat. Energy.

