# Supplementary material for: Chalcogen heteroatom engineering of solid additives with optimized excited-state properties enables the development of high-performance organic solar cells
Source: Chem Sci. 2026 Sep 11;17(42):20595–605. doi: 10.1039/d6sc04665g (PMC13587840; doi:10.1039/d6sc04665g)
Supplement: Supplementary file 1 [file SC-017-D6SC04665G-s001.pdf]

Supplementary Information (SI) for Chemical Science.
This journal is © The Royal Society of Chemistry 2026

Supporting Information

Chalcogen heteroatom engineering of solid additives with optimized excited-state properties enables high-performance organic solar cells

You-Dan Zhang [a,*], Xue Chen [a], Shun Zhang [b], Huan-Tao Xu [c], Rong-Rong Liu [a], Xin-Ru Gao [a], Haojie Du [d], Yuhan Song [d], Cheng-Long Wang [a], Chunfeng Zhang [c,*]

[a] National Green Coating Equipment and Technology Research Centre, Lanzhou Jiaotong University, Lanzhou 730070, China.

[b] Lanzhou Longneng Electric Power Science & Technology Ltd., Lanzhou 730070, China.

[c] National Laboratory of Solid State Microstructures, Collaborative Innovation Center of Advanced Microstructures, Nanjing University, Nanjing 210093, China.

[d] School of Environmental and Municipal Engineering, Lanzhou Jiaotong University, Lanzhou 730070, China.

1. Materials

Polymer donors (D18 and PM6) and non-fullerene acceptors (L8-BO and Y6) were purchased from Solarmer Material Inc. Cathode interface material (PDINN) was purchased from Derhon Optoelectronics Materials Science Technology Co LTD. Solid additives, benzofuran (BFZ) and 2,1,3-benzothiadiazole (BTD), were purchased from Aladdin. All the materials were directly used without any further purification.

2. Device fabrication

The organic solar cell (OSC) devices were fabricated with a conventional architecture of ITO/PEDOT:PSS/active layer/PDINN/Ag. First, the ITO-coated glass

was cleaned with deionized water, acetone and isopropyl alcohol under ultrasonication for 10 min sequentially. Next, the surface of the ITO-coated glass was exposed to UV-ozone for 10 min. PEDOT:PSS was deposited on the ITO substrate at 3000 rpm for 30 s and then dried at 155 °C for 15 min in air. The substrates were then moved into a glove box filled with nitrogen. The blend PM6:L8-BO (1:1.2, w/w) was dissolved in chloroform to a total mixed concentration of 16.5 mg/mL. 96 wt% of BFZ and BTD were added. All solutions were stirred at 60 °C for 2 to 3 hours and spin-coated onto the substrates at the speed of 2400-3500 rpm. Then all the active layers were subjected to thermal annealing at 100 °C for 10 min. Subsequently, PDINN (1 mg/mL) was spin-coated onto the active layer at 3000 rpm for 30 s, serving as the electron transporting layer. Finally, 100 nm Ag was deposited under vacuum pressure of 4×10^{-4} Pa onto the PDINN layer with a shadow mask. The effective area of the device was 3.2 mm^2.

3. Device characterizations

3.1 DFT calculation

Density functional theory (DFT) was performed Gaussian program package. Optimized structures of PM6 and L8-BO were calculated at the B3LYP/6-31G(d) level.

3.2 CA measurement

Contact angles (CA) were measured by a JY-PHa contact angle tester. Surface energy of the material could be divided into dispersive and polar components by Owens-Wendt method:

$$\gamma = \gamma^d + \gamma^p \quad (1)$$

Based on the contact angle of the two different solvents, the dispersion surface energy and the polar surface energy can be calculated by the following formula.

$$(1 + \cos\theta)\gamma_L = 2\sqrt{\gamma_S^d \gamma_L^d} + 2\sqrt{\gamma_S^p \gamma_L^p} \quad (2)$$

θ is the contact angle of solvent, γ_L is the surface energy of the solvent, γ_s^d and γ_s^p refer to the dispersive and polar surface energy of the solid, respectively, γ_L^d and γ_L^p refer to the dispersive and polar surface energy of the solvent, respectively. The Flory–Huggins interaction parameter can be calculated by the equation:

$$\chi_{a:b} = K\left(\sqrt{\gamma_a} - \sqrt{\gamma_b}\right)^2 \quad (3)$$

where γ_a and γ_b are the surface energy of materials a and b, respectively.

3.3 *J-V* and EQE characterizations

Photovoltaic performance was measured by a solar simulator (Newport) under AM 1.5 G spectroscopy with an effective area of 0.032 cm^2. The current density-voltage (*J-V*) characteristics were recorded by a Keithley 2400 light source meter with a range of -0.2 to 1.0 V, a scan step of 20 mV, and a delay time of 0.01 s. The light intensity of the light source was calibrated prior to the test using a standard silicon (Si) solar cell calibrated by a National Renewable Energy Laboratory (NREL) certified Si photodiode. A value of 100 mW/cm^2 was derived. The external quantum efficiency (EQE) spectra were obtained from a commercial EQE measurement system (QE-R3011, Enlitech, Taiwan). The light intensity at each wavelength was calibrated by a standard monocrystalline silicon photovoltaic cell.

3.4 SCLC measurement

Hole-only and electron-only devices were fabricated to measure the corresponding mobilities of active layers by using the space charge limited current (SCLC) method with the structure of ITO/PEDOT:PSS/active layer/MoO_3/Ag and ITO/ZnO/active layer/PDINN/Ag, respectively. Here, the preparation of active layer was consistent with that of the photovoltaic devices. The mobilities (μ) were determined by fitting the dark current to the model of a single carrier SCLC, described by the equation:

$$J = \frac{9}{8}\varepsilon_0\varepsilon_r\mu\frac{V^2}{d^3} \tag{4}$$

where ε_0 is the permittivity of the free space, $\varepsilon_r\approx 3$ is the average dielectric constant of the blend, μ is the charge carrier mobility, d is the thickness of the active layer, and V is the effective voltage. The effective voltage was obtained by subtracting the built-in voltage (V_{bi}, the built-in voltage due to the relative work function difference of the two electrodes) and the voltage drop (V_s, due to contact resistance and series resistance across the electrodes) from the applied voltage (V_{appl}), $V = V_{appl} - V_{bi} - V_s$. The mobility was calculated from the slope of the $J^{0.5}$-V curves. The thickness of the BHJ blend for SCLC measurement was about 100 nm.

3.5 AFM, TEM and GIWAXS characterizations

The atomic force microscopy (AFM) images were obtained using a Nano Scope NS3A system (Digital Instrument). The transmission electron microscope (TEM) images were investigated by transmission electron microscopes (Hitachi, HT7800). The grazing-incidence wide-angle X-ray scattering (GIWAXS) data were obtained at beamline BL02U2 of Shanghai Synchrotron Radiation Facility (SSRF). The

monochromaticity of the light source was 1.24 Å. The data were recorded by using the two-dimensional image plate detector of Pilatus 2M from Dectris, Switzerland.

3.6 TA spectroscopy measurement

Transient absorption (TA) spectroscopy measurement was conducted on a TA spectrometer (Ultrafast Systems, Helios Fire). The fundamental output pulse from a Ti: sapphire regenerative amplifier (800 nm, 35 fs, 1 kHz) was split into two beams. The pump beam was generated from an optical parametric amplifier (In this work, the wavelength of pump pulse was tuned at 800 nm to selectively excite the acceptor.); another beam was used to pump the calcium fluoride (CaF_2) crystal to generate the probe pulses. A mechanical chopper with a synchronized readout of a CMOS detector can capture the change in probe spectra, thus calculating the relative differential absorption (ΔT/T).

3.7 Voltage loss measurement

Fourier transform photocurrent spectroscopy (FTPS) was measured using an integrated system (PECT-600, Enlitech), where the photocurrent was amplified and modulated by a lock-in instrument. Electroluminescence external quantum efficiency (EQE_{EL}) was performed by applying external voltage/current sources through the devices (ELCT-3010, Enlitech). The voltage loss in solar cells can be attributed to the three factors shown in equation:

$$E_{\mathrm{loss}} = q\Delta V = E_{\mathrm{g}} - qV_{\mathrm{oc}}$$

$$= (E_{\mathrm{g}} - qV_{OC}^{SQ}) + (qV_{OC}^{SQ} - qV_{OC}^{rad}) + qV_{OC}^{rad} - qV_{\mathrm{oc}})$$

$$= (E_{\mathrm{g}} - qV_{OC}^{SQ}) + q\Delta V_{oc}^{rad,below\ gap} + q\Delta V_{oc}^{non-rad}$$

$$= \Delta E_1 + \Delta E_2 + \Delta E_3 \tag{5}$$

where q is the elementary charge, ΔV is the voltage loss, $\Delta V_{oc}^{rad,below\ gap}$ is the voltage loss of radiative recombination from the absorption below the bandgap, $\Delta V_{oc}^{non-rad}$ is the voltage loss of non-radiative recombination, V_{OC}^{SQ} is the maximum voltage according to the Shockley-Queisser limit, and V_{OC}^{rad} is the open-circuit voltage when there is only radiative recombination.

3.8 Calculation processes of exciton diffusion length

Exciton diffusion length can be determined by the exciton annihilation method. L_D was measured using pump fluence-dependent transient absorption (TA) spectroscopy. In general, the diffusion length of excitons can be evaluated using $L_D=(D\tau)^{0.5}$, where τ is the exciton lifetime and D is the diffusion constant. D is given by $D=\alpha/(8\pi R)$ (three-dimensional diffusion model), where α is the bimolecular exciton annihilation rate constant, R is the annihilation radius of excitons, taking the literature value of the annihilation radius (R = 1 nm).[1] The values of α and τ can be estimated from the experimental data of exciton decay dynamics considering the exciton-exciton annihilation (EEA). The initial exciton density $N(0)$ was estimated from the pump fluence and the optical density of the film at the excitation wavelength:

$$N(0) = \frac{F(1 - 10^{-OD})}{Ed} \tag{6}$$

where F is the pump photon fluence, OD is the optical density of the film at the pump wavelength, E is single photon energy and d is the film thickness.

$$E = h\frac{c}{\lambda} \tag{7}$$

where h is Planck's constant, c is the speed of light, and λ is the pump

wavelength.

The exciton decay dynamics characterized by TA spectroscopy can be described approximately as:

$$N(t) = \frac{N(0)e^{-kt}}{1 + \frac{\alpha}{2k}N(0)[1 - e^{-kt}]} \tag{8}$$

where $k=1/\tau$ is the intrinsic exciton decay rate constant, and $N(t)$ is the exciton density as a function of time delay after excitation. **Fig. S9** shows the fluence-dependent decay traces of photon-induced excitons in L8-BO (w/o), L8-BO (BFZ), and L8-BO (BTD) films, respectively. The decays of the excitons are strongly dependent on the excitation intensity. The related parameters are summarized in **Table S10**.

4. The universality of solid additives

In order to examine the universality of BTD, devices were fabricated processed with BTD in different donor/acceptor systems, including PM6:Y6 and D18:L8-BO. The chemical structures of Y6 and D18 are shown in **Fig. S4**. The *J-V* curves of devices based on different blend systems are displayed in **Fig. S5**, and the corresponding parameters are summarized in **Table S4**. The as-cast OSCs based on PM6:Y6 and D18:L8-BO exhibit PCEs of 16.35% and 17.35%, respectively. Surprisingly, after BTD processing, the PCEs of the OSCs were increased to 17.13% and 18.49%, respectively.

Notably, the PCE values of all BTD-based devices exceeded those of devices prepared under conventional optimization conditions. This indicates that BTD

exhibits decent universality in multiple material systems, giving the strategy considerable potential for optimizing intermolecular interaction, properties of excited states and improving the performance of OSCs.

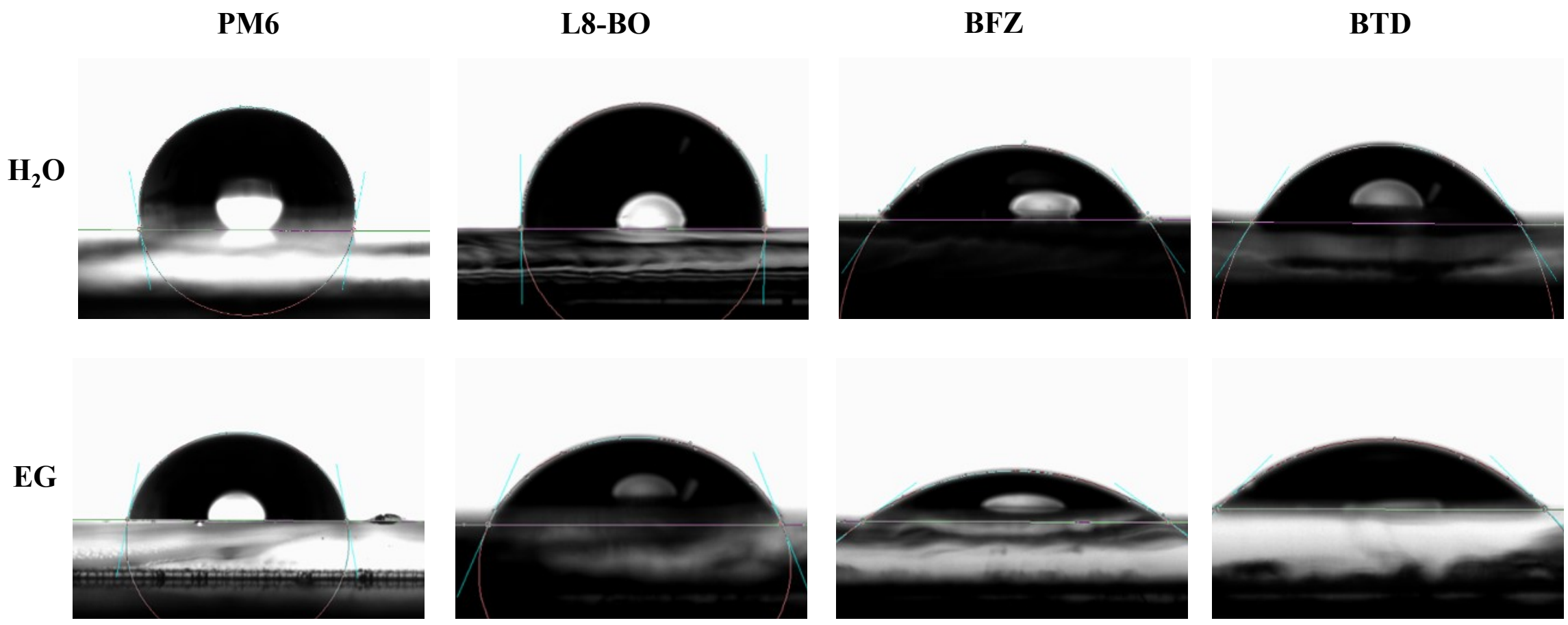

Fig. S1 Contact angle images of various films with water and ethylene glycol drop on top.

Table S1 Detailed data for contact angle.

	θ_{H_2O} (°)	θ_{EG} (°)	γ^d (mN/m^2)	γ^p (mN/m^2)	γ (mN/m^2)	χ (BFZ)	χ (BTD)
BFZ	49.5	30.4	2.70	68.53	71.23	-	-
BTD	51.7	40.3	6.59	43.29	49.88	-	-
PM6	100.5	78.8	17.33	2.11	19.43	16.25	7.05
L8-BO	91.2	66.0	22.71	3.53	26.24	11.00	3.76

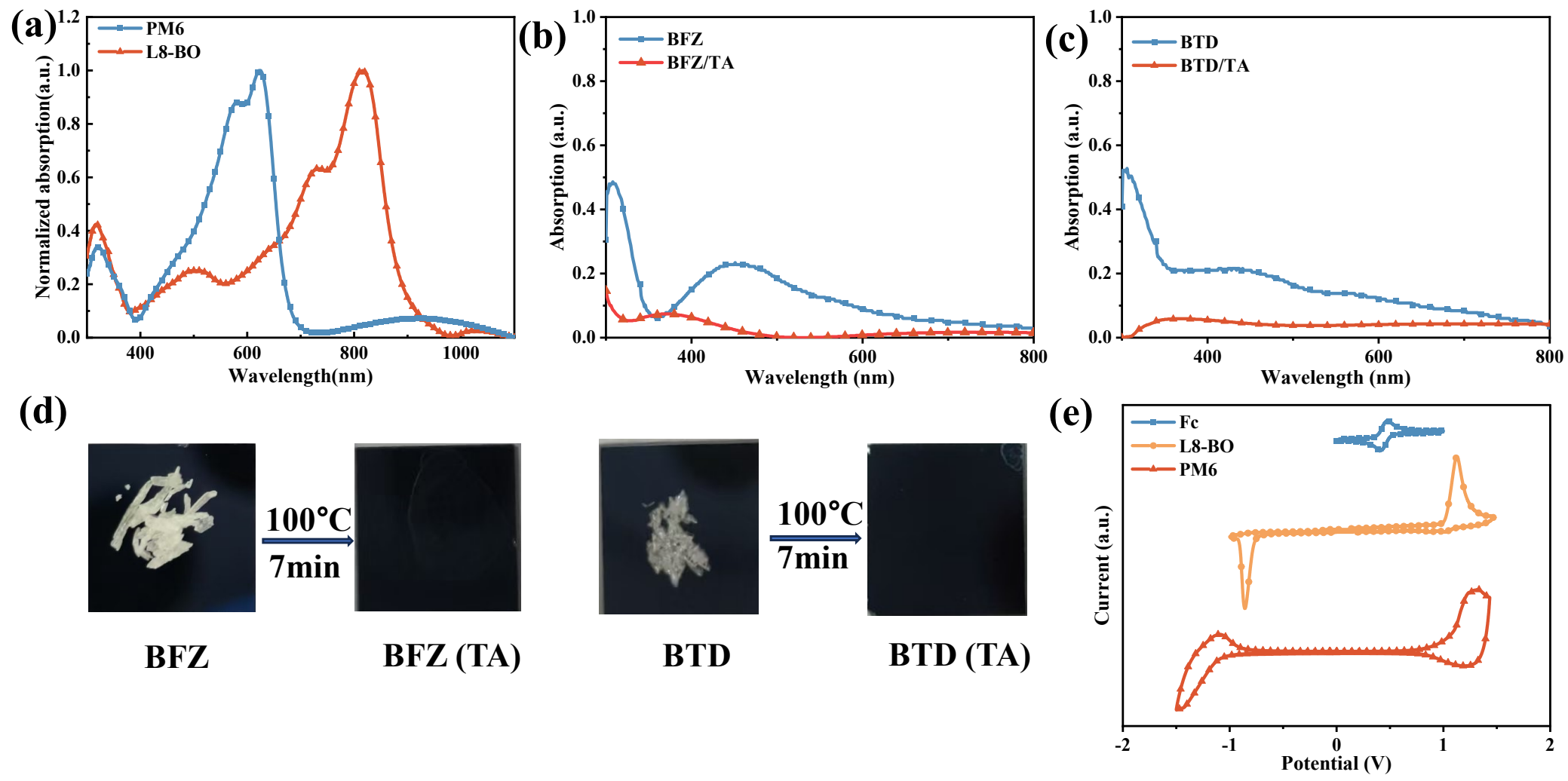

Fig. S2 (a) UV-Vis absorption spectra of pure films of PM6 and L8-BO. UV-Vis absorption spectra of pure films of (b) BFZ and (c) BTD before and after annealing. (d) BFZ and BTD on a silicon wafer and kept at 100 °C for a certain time. (e) CV diagram of Fc, PM6 and L8-BO.

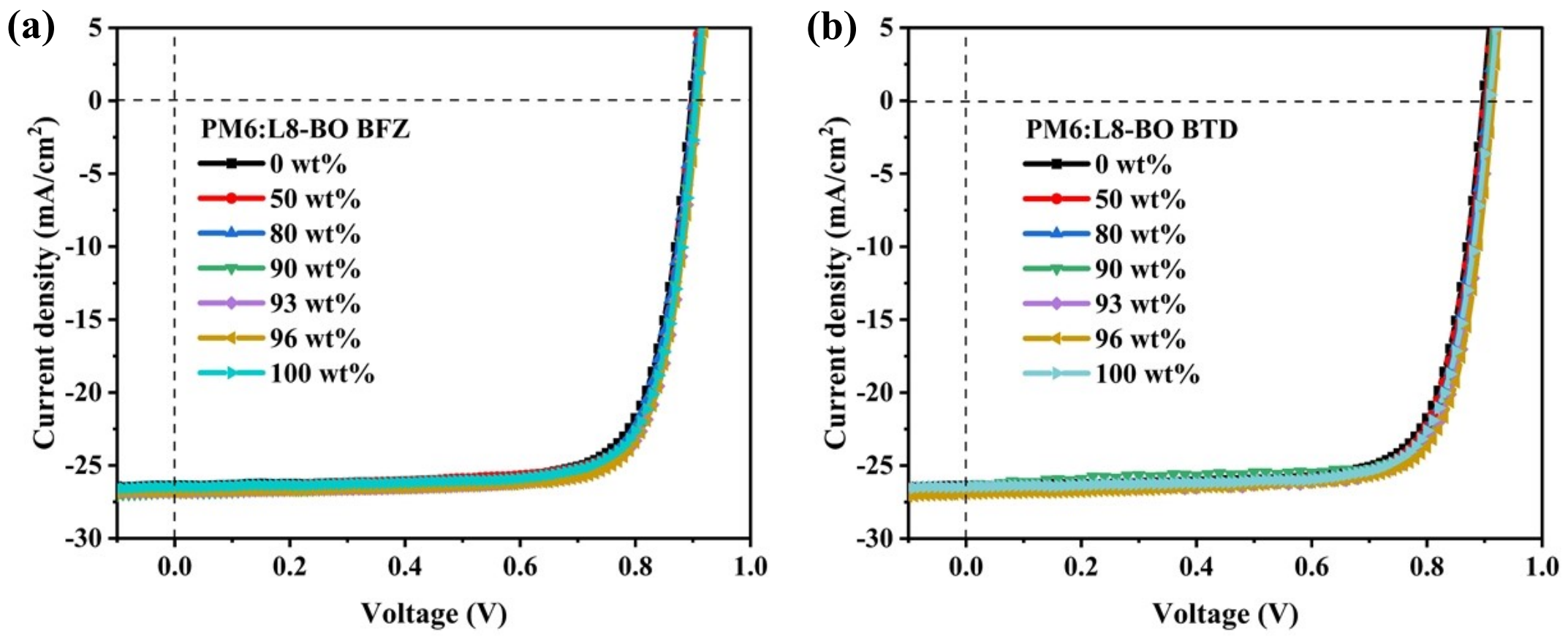

Fig. S3 *J-V* curves of the OSCs based on PM6:L8-BO processed with different additive concentrations of (a) BFZ and (b) BTD, respectively.

Table S2 Photovoltaic parameters of the OSCs based on PM6:L8-BO processed with different additive concentrations of BFZ.

PM6:L8-BO	V_{oc} (V)	J_{SC} (mA/cm^2)	FF (%)	PCE (%)
BFZ (0 wt%)	0.900	26.36	76.58	18.12
BFZ (50 wt%)	0.900	26.55	77.17	18.46
BFZ (80 wt%)	0.901	26.84	76.53	18.50
BFZ (90 wt%)	0.904	26.83	77.62	18.83
BFZ (93 wt%)	0.906	26.80	77.85	18.90
BFZ (96 wt%)	0.910	26.83	77.99	19.04
BFZ (100 wt%)	0.906	26.46	77.12	18.49

Table S3 Photovoltaic parameters of the OSCs based on PM6:L8-BO processed with different additive concentrations of BTD.

PM6:L8-BO	V_{oc} (V)	J_{SC} (mA/cm^2)	FF (%)	PCE (%)
BTD (0 wt%)	0.900	26.36	76.58	18.12
BTD (50 wt%)	0.902	26.59	76.85	18.43
BTD (80 wt%)	0.905	26.63	77.18	18.60
BTD (90 wt%)	0.906	26.70	77.44	18.73
BTD (93 wt%)	0.910	26.81	77.61	18.93
BTD (96 wt%)	0.915	26.91	77.64	19.11
BTD (100 wt%)	0.909	26.45	77.03	18.52

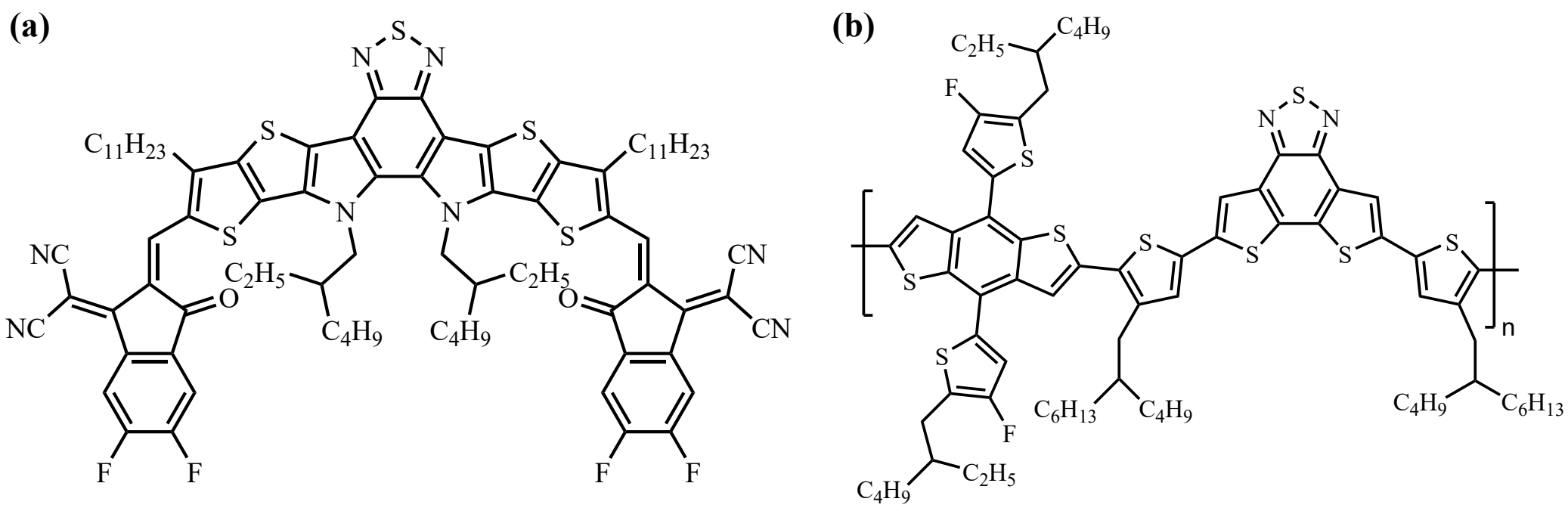

Y6 D18

Fig. S4 The molecular structures of (a) Y6 and (b) D18.

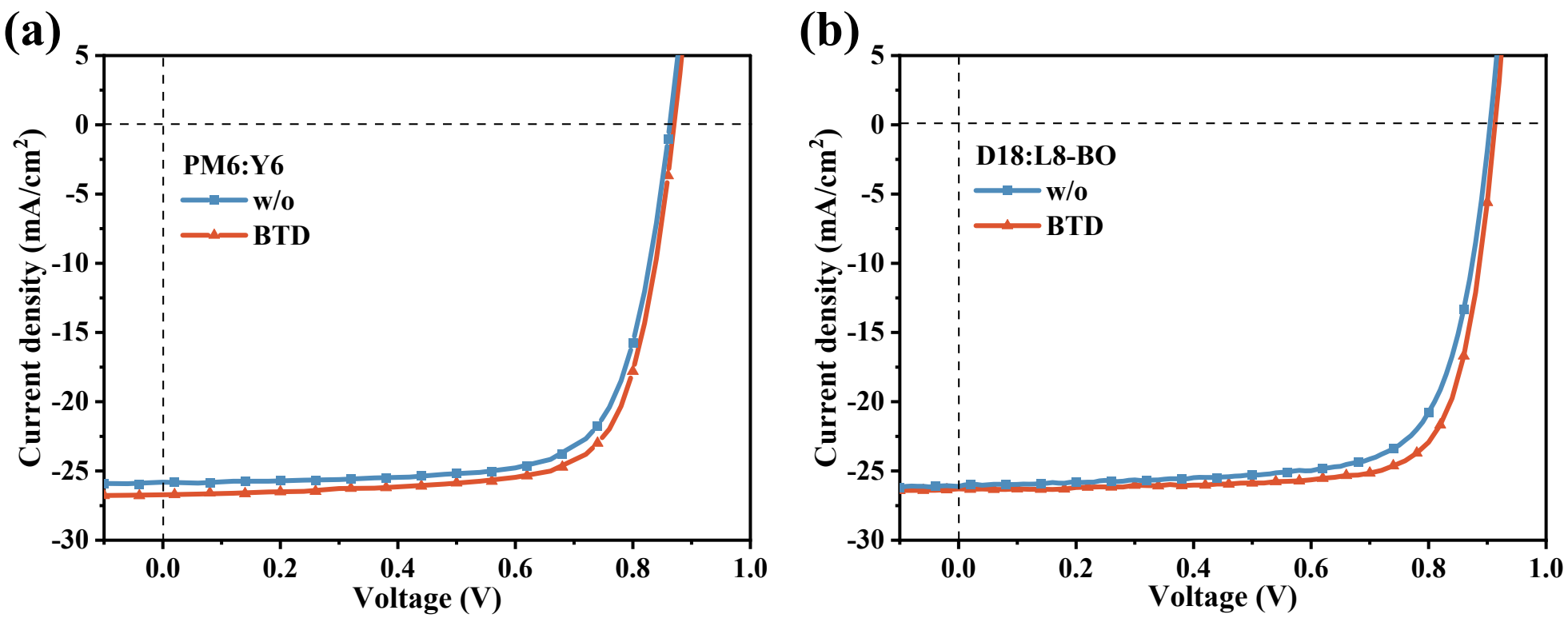

Fig. S5 *J-V* curves in different OSCs systems including (a) PM6:Y6-based devices, and (b) D18:L8-BO-based devices.

Table S4 The photovoltaic parameters of PM6:Y6 and D18:L8-BO based the control and with BTD devices, respectively.

Active layer	V_{oc} (V)	J_{SC} (mA/cm^2)	FF (%)	PCE (%)
PM6:Y6 (w/o)	0.863	25.81	73.42	16.35
PM6:Y6 (BTD)	0.870	26.67	73.70	17.13
D18:L8-BO (w/o)	0.905	26.11	73.44	17.35
D18:L8-BO (BTD)	0.913	26.30	77.03	18.49

Table S5 Exciton dissociation and charge collection probability of the PM6:L8-BO-based devices.

PM6:L8-BO	P_{diss} (%)	P_{coll} (%)
w/o	95.68	84.50
BFZ	97.73	86.11
BTD	98.37	89.55

Table S6 J_{sc}-P_{light} and V_{oc}-P_{light} details of PM6:L8-BO-based devices.

PM6:L8-BO	α	$n\ k_B T/q$
w/o	0.991	1.26
BFZ	0.994	1.22
BTD	0.997	1.20

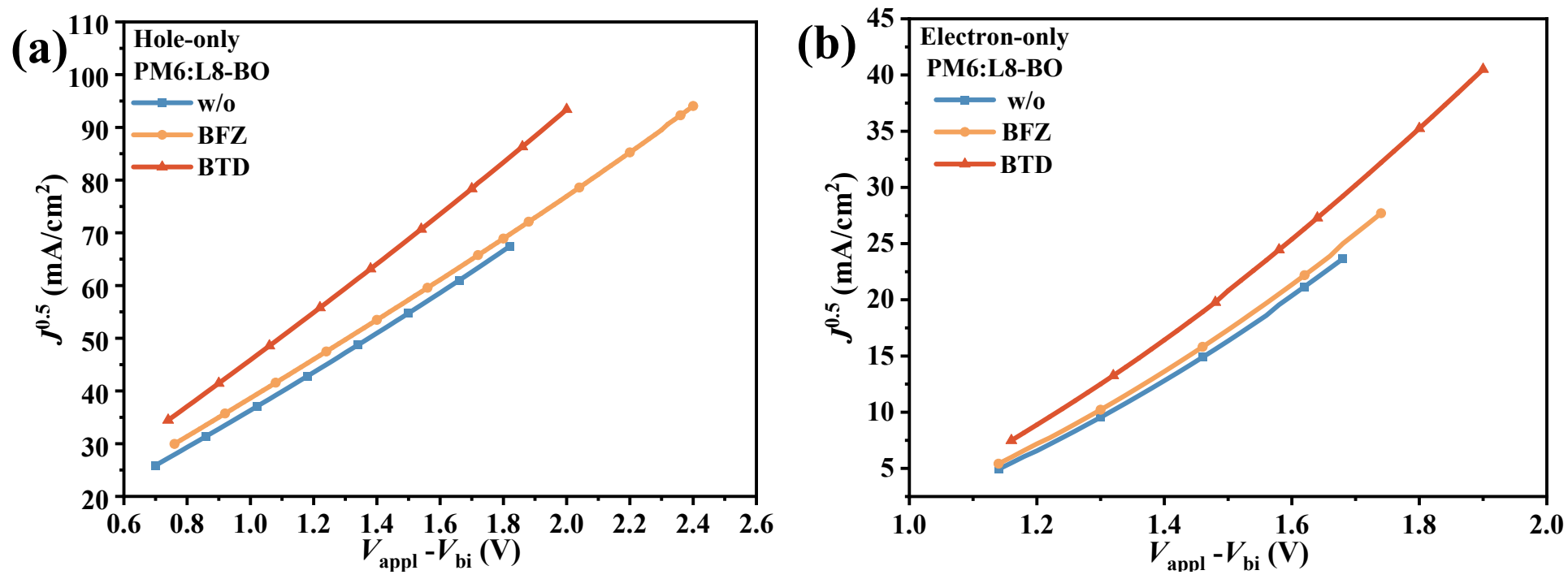

Fig. S6 The $J^{0.5}$-V plots of (a) hole-only devices and (b) electron-only devices.

Table S7 Hole and electron mobility.

PM6:L8-BO	μ_h ($\times 10^{-4}$ cm^2 V^{-1} s^{-1})	μ_e ($\times 10^{-4}$ cm^2 V^{-1} s^{-1})	μ_h/μ_e
w/o	4.59	4.01	1.14
BFZ	5.08	4.58	1.11
BTD	7.30	6.74	1.09

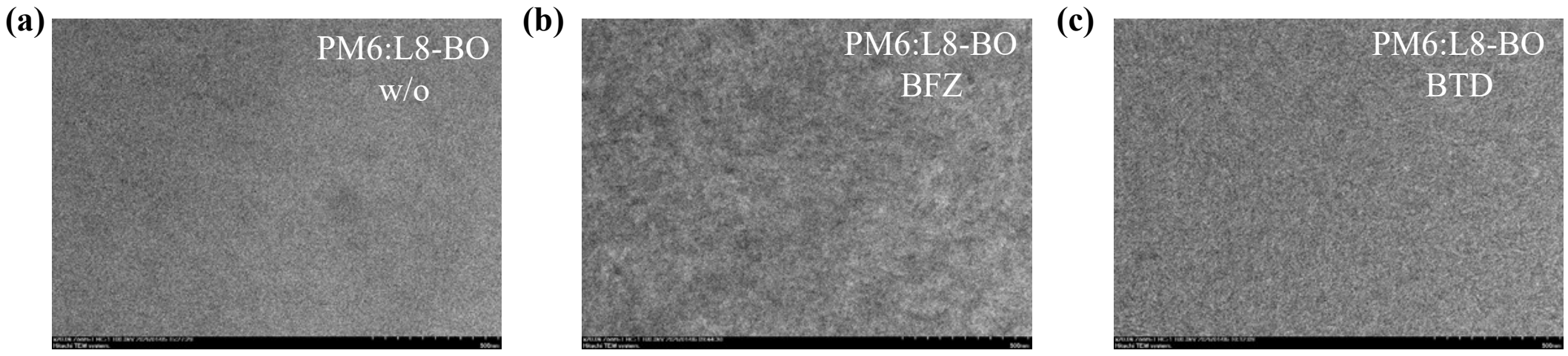

Fig. S7 TEM images of (a) without, (b) with BFZ and (c) with BTD-processed PM6:L8-BO blend films.

Table S8 Summary of detailed GIWAXS data of (010) peak in OOP.

OOP	q_z (Å^{-1})	FWHM (Å^{-1})	d-spacing (π-π) (Å)[a]	CCL (Å)[b]
w/o	1.61	0.25	3.91	22.45
BFZ	1.61	0.24	3.90	23.72
BTD	1.62	0.23	3.88	24.45

[a] d-spacing =$2\pi/q$; [b] CCL =$2\pi K$/FWHM, where K is a shape factor (K=0.89)

Table S9 Summary of detailed GIWAXS data of (100) peak in IP.

IP	q_{xy} (Å^{-1})	FWHM (Å^{-1})	d-spacing (lamellar) (Å)[a]	CCL (Å)[b]
w/o	0.31	0.10	20.47	57.68
BFZ	0.31	0.09	20.44	63.80
BTD	0.31	0.09	20.40	64.24

[a] d-spacing =$2\pi/q$; [b] CCL =$2\pi K$/FWHM, where K is a shape factor (K=0.89)

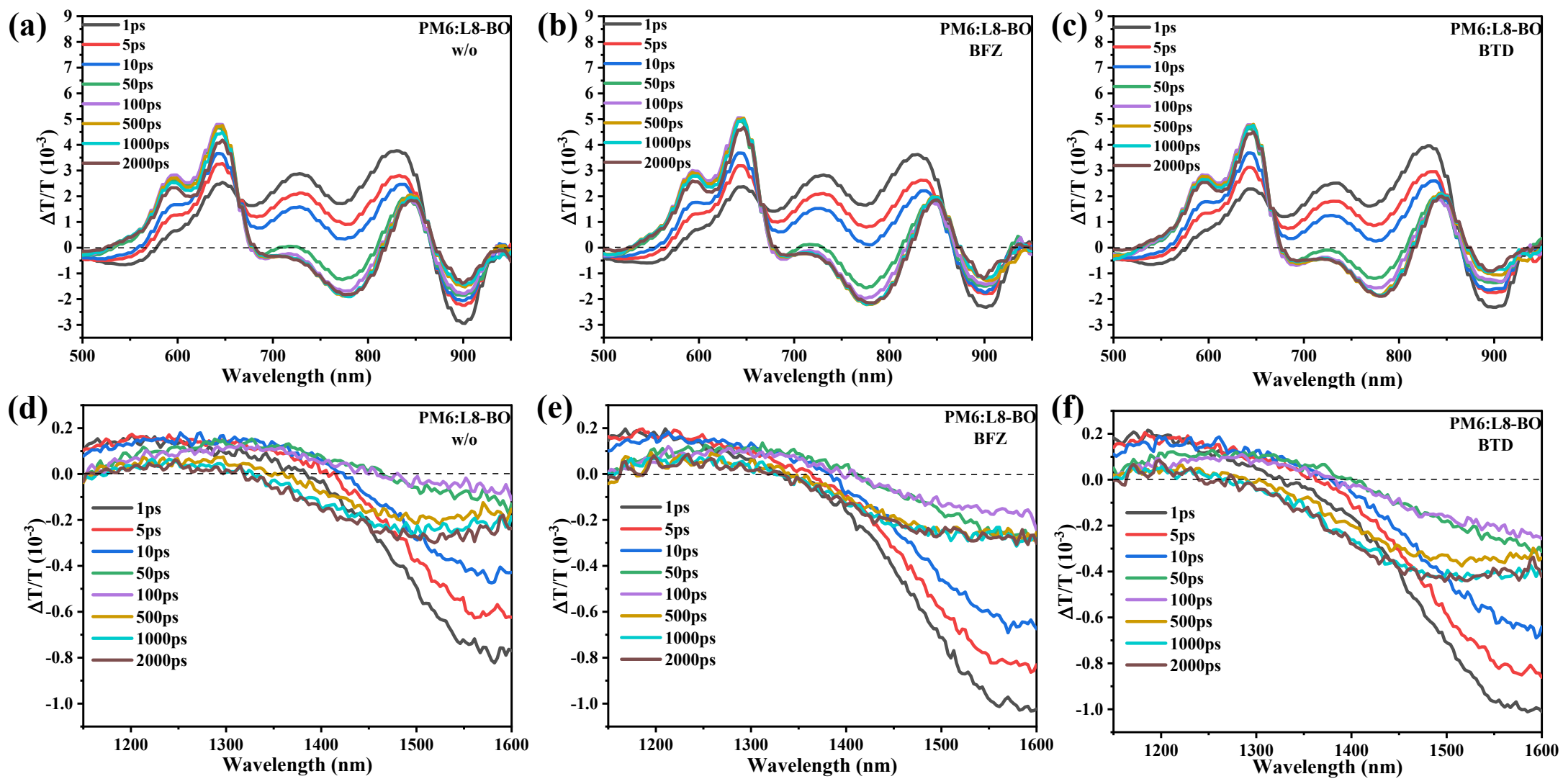

Fig. S8 The TA absorption curves as a function of delay time for PM6:L8-BO blend film (a, d) without, (b, e) with BFZ, and (c, f) with BTD.

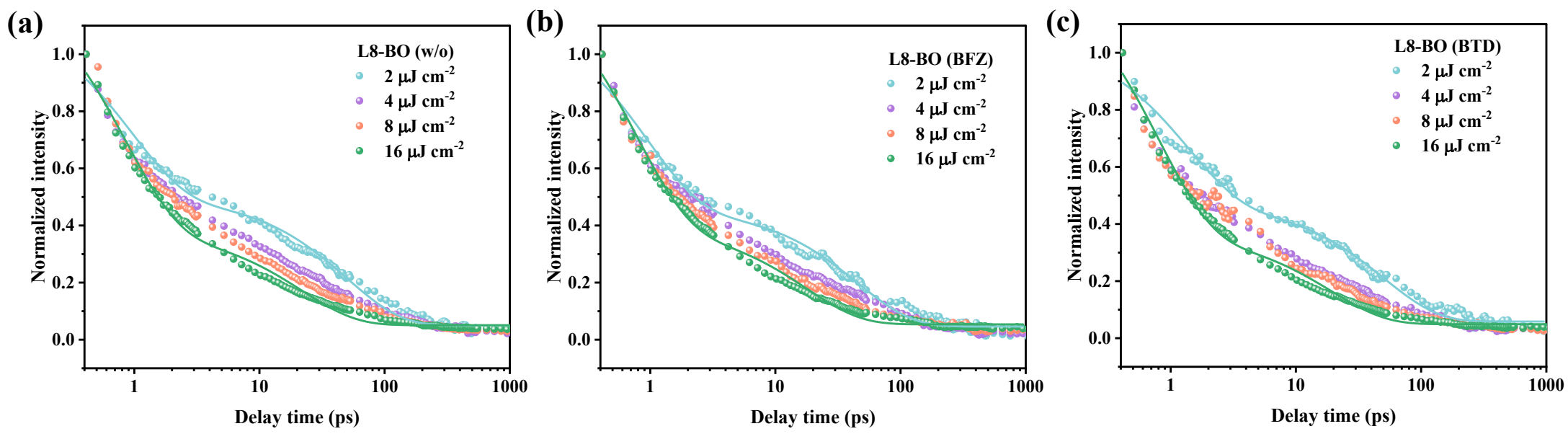

Fig. S9 Decay dynamics of (a) without, (b) BFZ-treated and (c) BTD-treated L8-BO films.

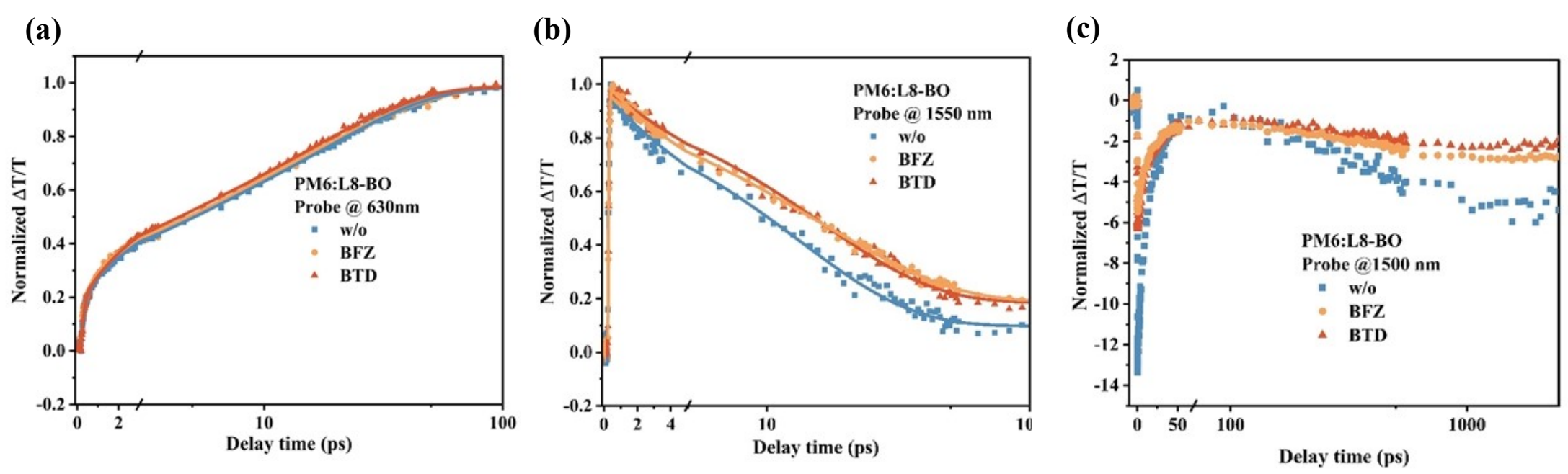

Fig. S10 Normalized TA kinetics probed at (a) 630 nm, (b) 1550 nm and (c) 1500 nm.

Table S10 Fitting parameters for the pump fluence-dependent TA decay curves.

Films	τ (ps)	k (s^{-1})	α (cm^3/s)	D (cm^2/s)	L_D (nm)
L8-BO (w/o)	336.7	2.97×10^{9}	2.60×10^{-8}	1.04×10^{-2}	18.7
L8-BO (BFZ)	305.0	3.28×10^{9}	4.80×10^{-8}	1.91×10^{-2}	24.1
L8-BO (BTD)	297.1	3.37×10^{9}	6.06×10^{-8}	2.41×10^{-2}	26.7

Table S11 Fitting parameters extracted from TA measurements of the control and BFZ-, BTD-treated PM6:L8-BO blend films.

PM6:L8-BO (630 nm)	τ_1 (ps)	A_1	τ_2 (ps)	A_2
w/o	0.38±0.06	0.24±0.02	21.44±1.71	0.51±0.03
BFZ	0.19±0.02	0.39±0.04	19.81±0.69	0.57±0.01
BTD	0.13±0.01	0.67±0.12	17.35±0.64	0.57±0.01

Table S12 Detailed energy losses in devices with and without additives.

PM6: L8-BO	E_g (eV)	qV_{OC}^{SQ} (eV)	ΔE_1 (eV)	qV_{oc}^{rad} (eV)	ΔE_2 (eV)	EQE_{EL} (%)	ΔE_3 (eV)	qV_{oc} (V)	E_{loss} (eV)	E_u (meV)
w/o	1.458	1.188	0.270	1.157	0.031	3.80×10^{-3}	0.263	0.900	0.564	24.05
BFZ	1.457	1.189	0.268	1.159	0.029	6.22×10^{-3}	0.250	0.910	0.547	23.40
BTD	1.457	1.189	0.268	1.161	0.028	7.30×10^{-3}	0.246	0.915	0.542	23.19

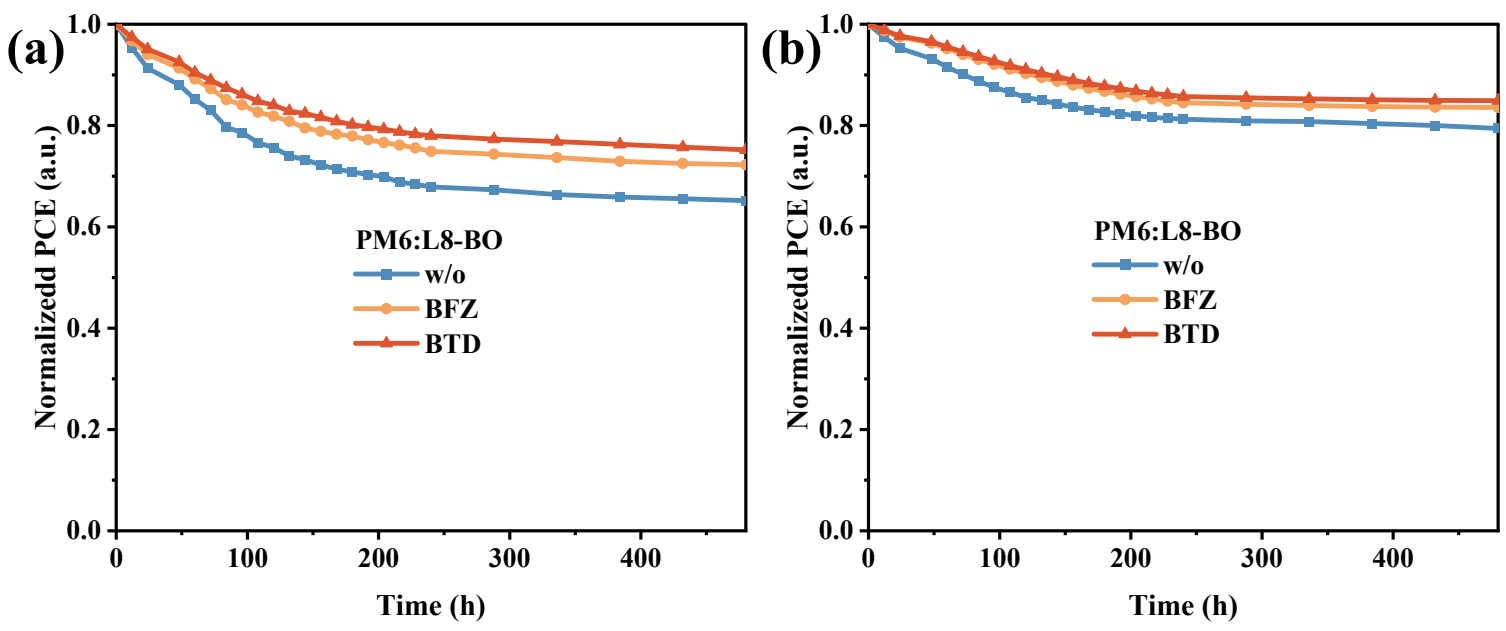

Fig. S11 (a) Thermal stability and (b) storage stability of normalized PCE based on

without, with BFZ and with BTD devices in a nitrogen glovebox.

References

1 D. Luo, Z. Jiang, W. Tan, L. Zhang, L. Li, C. Shan, C. McNeill, P. Sonar, B. Xu and A. Kyaw, Non-fused ring acceptors achieving over 15.6% efficiency organic solar cell by long exciton diffusion length of alloy-like phase and vertical phase separation induced by hole transport layer, *Adv. Energy Mater.*, 2023, **13**, 2203402.
